# Age-related decline in the resistance of mice to bacterial infection and in LPS/TLR4 pathway-dependent neutrophil responses

**DOI:** 10.3389/fimmu.2022.888415

**Published:** 2022-08-24

**Authors:** Kirsti Hornigold, Julia Y. Chu, Stephen A. Chetwynd, Polly A. Machin, Laraine Crossland, Chiara Pantarelli, Karen E. Anderson, Phillip T. Hawkins, Anne Segonds-Pichon, David Oxley, Heidi C. E. Welch

**Affiliations:** ^1^ Signalling Programme, The Babraham Institute, Cambridge, United Kingdom; ^2^ Bioinformatics Facility, The Babraham Institute, Cambridge, United Kingdom; ^3^ Proteomics Facility, The Babraham Institute, Cambridge, United Kingdom

**Keywords:** aging, neutrophils, TLR4, proteomics, ROS, degranulation, phagocytosis, NETs

## Abstract

Host defense against bacterial and fungal infections diminishes with age. In humans, impaired neutrophil responses are thought to contribute to this decline. However, it remains unclear whether neutrophil responses are also impaired in old mice. Here, we investigated neutrophil function in old mice, focusing on responses primed by lipopolysaccharide (LPS), an endotoxin released by gram-negative bacteria like *E. coli*, which signals through toll-like receptor (TLR) 4. We show that old mice have a reduced capacity to clear pathogenic *E. coli* during septic peritonitis. Neutrophil recruitment was elevated during LPS-induced but not aseptic peritonitis. Neutrophils from old mice showed reduced killing of *E. coli*. Their reactive oxygen species (ROS) production was impaired upon priming with LPS but not with GM-CSF/TNFα. Phagocytosis and degranulation were reduced in a partially LPS-dependent manner, whereas impairment of NET release in response to *S. aureus* was independent of LPS. Unexpectedly, chemotaxis was normal, as were Rac1 and Rac2 GTPase activities. LPS-primed activation of Erk and p38 Mapk was defective. PIP_3_ production was reduced upon priming with LPS but not with GM-CSF/TNFα, whereas PIP_2_ levels were constitutively low. The expression of 5% of neutrophil proteins was dysregulated in old age. Granule proteins, particularly cathepsins and serpins, as well as TLR-pathway proteins and membrane receptors were upregulated, whereas chromatin and RNA regulators were downregulated. The upregulation of CD180 and downregulation of MyD88 likely contribute to the impaired LPS signaling. In summary, all major neutrophil responses except chemotaxis decline with age in mice, particularly upon LPS priming. This LPS/TLR4 pathway dependence resolves previous controversy regarding effects of age on murine neutrophils and confirms that mice are an appropriate model for the decline in human neutrophil function.

## Introduction

Neutrophils provide the first line of innate host defense against bacterial and fungal infections (1), as well as playing roles in wound healing and cancer progression (2, 3). During inflammation and infections, neutrophils are rapidly recruited from the blood stream into the inflamed or infected tissue. They kill pathogens by phagocytosis, degranulation, reactive oxygen species (ROS) and neutrophil extracellular traps (NETs) (1).

Host defense against bacterial and fungal infections diminishes with age. In the elderly, infections are more likely to develop into dangerous diseases such as pneumonia and peritonitis, progress to sepsis, and cause death from sepsis (4, 5). In humans, the age-related decline in neutrophil function is thought to contribute to the decreased immunity against bacteria and fungi, although a causal relationship is hard to establish. The elderly have normal numbers of circulating neutrophils (6), but all major neutrophil responses are altered. ROS and NET production are impaired in response to a wide range of stimuli (7–10), chemotaxis is reduced under multiple conditions, and chemokinesis is impaired upon TNFα priming (8, 11). The reduced chemotaxis has been linked to constitutively increased PI3K activity, where inhibition of PI3Kγ or PI3Kδ restored migration speed and accuracy (11). Degranulation of azurophil granules seems increased in old age (11), whereas phagocytosis is decreased (12, 13). These altered neutrophil responses have largely been attributed to the increased inflammatory state associated with advanced age (inflamm-aging), where chronically increased levels of a range of inflammatory mediators perturb signaling pathways. Impairments in signaling through the inflammatory cytokines GM-CSF and TNFα, and through toll-like receptor (TLR) pathways, are commonly reported (14). For example, the GM-CSF-mediated delay in neutrophil apoptosis which occurs during inflammation and infections is reduced in the elderly (8), as is the TNFα-priming of chemokinesis and chemotaxis (11). Neutrophils from the elderly express normal levels of TLR1, TLR2 and TLR4 (6, 8, 10). However, the cell surface level of TLR1 is reduced, and the TLR1-mediated upregulation of Mac1, shedding of L-selectin and production of IL8 are impaired (6), as is the LPS-stimulated recruitment of TLR4 into lipid rafts (8).

In mice, immunity against bacterial and fungal pathogens declines with age just as in humans. For example, old mice are less able to clear *Staphylococcus aureus* from skin wounds, leading to delayed wound closure (15), or to resolve *Salmonella typhimurium* after oral infection, causing the colonization of multiple organs (16), or clear *Candida albicans* after intravenous infection, leading to high fungal tissue burden and mortality (17). Old mice also show increased mortality after infection with influenza or *Herpes simplex* virus, and neutrophil depletion revealed that these losses of antiviral immunity are neutrophil-dependent (18, 19). Considering these consistent reductions in the immunity of old mice to a range of pathogens, the effects of age on neutrophil recruitment to infected tissues are surprisingly varied. Neutrophil recruitment to the skin was reduced in old mice after *S. aureus* infection (15), whereas peritoneal recruitment was increased during *C. albicans* infection (20), and recruitment to the lung was delayed during infection with *Francisella tularensis* (21) but increased during influenza virus infection (19). When neutrophil recruitment was induced with various inflammatory mediators, the effects of aging were similarly inconsistent. Keratinocyte-derived chemokine (KC)-induced neutrophil recruitment to the skin was reduced in old mice (15), as was IL-1 induced recruitment to the cremaster muscle (22), whereas LPS-induced recruitment to the lung was increased (23). Therefore, the age-related decline in immunity is clearly not simply correlated with neutrophil recruitment, suggesting that neutrophil effector responses may be reduced in old age. However, reports on the effects of age on mouse neutrophil responses show great variability. ROS production was reduced in neutrophils from old mice upon stimulation with TLR2 ligands (24), but normal in response to the oxysterol 7KC (25). NETosis was reduced in neutrophils from old mice upon stimulation with TLR2 ligands (24), but increased upon mitochondrial oxidative stress (25). Furthermore, despite neutrophil transcriptome analysis showing altered expression of NETosis-related genes with age, including histones, PADI4 and elastase, PMA-induced NETosis was normal (26). Phagocytosis of yeast by neutrophils from old mice was reduced (27), whereas phagocytosis of *S. aureus* was normal (15). Transwell assays showed either normal or increased spontaneous migration of neutrophils from old mice, but a loss of KC-induced chemotaxis (19, 28). These variabilities have led to the proposal that the age-related decline in murine neutrophil responses is too subtle and complex for mice to be a useful model for the decline in human neutrophil function (29).

Over recent years, research focus has therefore shifted from studying the influence of organismal age on neutrophil function to investigating the aging of individual neutrophils. This showed that neutrophils age between their release from the bone marrow into the circulation and their homeostatic clearance into tissues a few hours or at most days later. In both humans and mice, neutrophils freshly released into the circulation have high surface levels of L-selectin (CD62L) which decline over several hours, whereas aged neutrophils show increased CXCR4 surface levels before they are cleared from the blood (30–32). Fresh neutrophils are preferentially recruited to inflamed and infected organs to provide host defense, whereas the homeostatic clearance of aged neutrophils serves to protect against vascular inflammation and patrol for pathogens in the homing tissues (30). The mechanism underlying this neutrophil aging is that the cells become primed by signaling through TLRs during their time in the circulation (31, 32). Elegant experiments with germ-free mice have shown that the stimuli responsible for this priming are the TLR4 ligand LPS and TLR2 ligand peptidoglycan released by the gut microbiota (32). Unlike tissue recruitment however, the capacity of neutrophils to produce ROS and cytokines is unaltered throughout their aging in the circulation (30, 31).

Intrigued by the discrepancies between previous studies on neutrophil responses in old mice, we sought to revive research efforts into the effects of organismal age on neutrophil function, focusing on the priming pathways that had been implicated in aging human neutrophils. We found an age-related decline in murine neutrophil function that is largely dependent on the LPS/TLR4 signaling pathway, thus resolving some of the previous controversy.

## Materials and methods

### Materials

Kwik-Diff stain (9990700) was from Thermo Scientific Shandon. Reagents for priming neutrophils were LPS (*E. coli*-derived, Sigma, L3024), mouse TNFα (R&D Systems, 410-MT-010), and mouse GM-CSF (Peprotech, 315-03). Reagents for stimulating neutrophils included f-Met-Leu-Phe (fMLP, Sigma, F3506), C5a (Sigma, C5788), and phorbol 12-myristate 13-acetate (PMA, Sigma, P1585). Antibodies for western blotting were Rac1 (clone 23A8, Millipore, 05-389, 1:3000), Rac2 (Millipore, 07-604, 1:5,000), Irak4 (Cell Signaling Technology, 4363, 1:500), MyD88 (Cell Signaling Technology, 4283, 1:500), TLR4 (Cell Signaling Technology, 14358, 1:500), and Trif (Abcam, ab13810, 1:1,000). Antibodies for LPS/TLR4 pathway analysis were from Cell Signaling Technology: phospho-p38 Mapk Thr180/Tyr182 (9211, 1:1000), p38 Mapk (9212, 1:1000), phospho-p42/44 Erk Thr202/Tyr204 (9106, 1:1000), p42/44 Erk (9102, 1:1000), phospho-Akt Thr308 (9275, 1:5000), and Akt (9272, 1:1000). Antibodies for other applications are listed in the relevant sections below. Fc block (553141) was from BD Biosciences.

### Mice

Male wild type mice on C57BL/6 genetic background at 8-10 weeks of age (young) and at 24 months of age (old) were compared directly in experiments. Mice were bred and group-housed (up to 5) in individually ventilated cages in the Babraham Institute Small Animal Facility that uses 12 h light/dark cycles with dusk and dawn settings, 52% room humidity (55 ± 10% range) and 20°C room temperature (19-21°C range), and were fed chow diet and water *ad libitum*. The C57BL/6 strain was originally purchased from Charles River (Margate, UK) and imported into the facility by embryo transfer. The unit has Specific Pathogen Free (SPF) status, monitored by quarterly testing of sentinels for 62 pathogens, exceeding current FELASA guidelines (33). Staff work in designated units, with showering-in, wearing of fresh autoclaved uniforms, gloves, hairnets and masks, and with a 48 h exclusion from other units. All materials are autoclaved or treated with vapourized hydrogen peroxide on import. The animal diet is sterilised to ≥25 Gy. Cages are opened in laminar flow cabinets. For infection with pathogenic *E. coli*, animals were housed in individually ventilated isocages in the Babraham Institute biosafety level 2 containment facility. Animal breeding and experiments were carried out with approval from the local Animal Welfare Ethical Review Body under the British Home Office Animal Scientific Procedures Act 1986.

### Peritonitis

Pathogenic *E. coli* O18:K1 bacteria (34) were used to induce septic peritonitis as described (35). The bacteria were grown in a biosafety level 2 facility to mid-log phase in Luria broth (LB) at 37°C, 5% CO_2_, pelleted, snap-frozen in PBS/20% glycerol and stored in aliquots at −80°C. Titers were determined by CFU count on blood agar plates. Prior to infection, a fresh aliquot of bacterial stock was thawed, washed in ice-cold Dulbecco’s Phosphate Buffered Saline (DPBS) without Ca^2+^ and Mg^2+^ (DPBS^–^, Sigma, D8537) by centrifugation at 10,000 × g for 2 min at 4°C, resuspended at 5 × 10^4^ bacteria/ml in ice-cold DPBS^–^, kept on ice and used within 1.5 h. To induce septic peritonitis, mice in a biosafety level 2 containment facility were injected *i.p.* with 200 μl of the *E. coli* suspension (1 × 10^4^ bacteria per animal), or were mock-treated with DPBS^–^, before being returned to their home cages with food and water *ad libitum*. 3 h later, mice were euthanized by CO_2_ asphyxiation, death confirmed by pithing, and peritoneal lavages performed by *i.p.* injection and aspiration of 8 mL DPBS^–^, 5 mM EDTA. A second lavage was performed, pooled with the first, and samples stored on ice. An aliquot of the lavage fluid was taken for enumeration of bacteria, serially diluted in ice-cold DPBS^–^, plated onto blood agar plates, cultured overnight at 37°C, 5% CO_2_, and CFU counted on plates of comparable density (20-200 bacteria). The remaining lavage cells were pelleted at 450 x g for 10 min at 4°C, erythrocytes lysed by resuspending cells in 1 ml Geye’s solution (130 mM NH_4_Cl, 5 mM KCl, 780 µM Na_2_HPO_4_, 176 µM KH_2_PO_4_, 5.5 mM glucose, 1 mM MgCl_2_, 280 µM MgSO_4_, 1.54 mM CaCl_2_, 13.4 mM NaHCO_3_) and incubated at RT for 150 s, prior to the addition of 10 ml DPBS with Ca^2+^ and Mg^2+^ (DPBS^++^, Thermo Fisher Scientific, 14040117) supplemented with 0.1% glucose and 4 mM NaHCO_3_ (DPBS^++++^). Leukocytes were centrifuged again and resuspended in 1 ml fixation buffer (Biolegend, 420801), incubated for 20 min at RT, washed again, and resuspended in 1 ml DPBS^++++^. Aliquots of fixed cells were counted by hemocytometer and the rest cytospun onto glass coverslips, stained with Kwik-Diff and imaged on a Zeiss Microbeam system. Leukocytes were enumerated taking into account the lavage volume recovered.

Aseptic peritonitis experiments were done essentially as previously described (36). Mice were injected *i.p.* with 0.25 ml sterile 3% thioglycollate (TGC, Sigma, T9032) in H_2_O, or were mock-treated with H_2_O. 3 h later, mice were euthanized and peritoneal lavages performed and erythrocytes lysed as described above. Leukocytes were resuspended in 1.25 ml DPBS^++++^. 1 ml of the sample was processed for flow cytometry by staining leukocytes with AF647-Cd11b (clone M1/70, BD Pharmingen, 557686, 1:800) and FITC-Gr1 (clone RB6-8C5, BD Pharmingen, 553126, 1:800) antibodies in DPBS^++++^ with Fc block for 20 min on ice in the dark, washing in DPBS^++++^, 5 mM EDTA and resuspension in 500 μl DPBS^++++^ containing 1 μg/ml DAPI and 1.25 × 10^5^ Spherotech ACBP-50-10 standard beads/ml (5.0-5.9 μm). Flow cytometry was carried out in a BD Biosciences LSRII flow cytometer, and FlowJo was used for data analysis. Neutrophils were identified by Cd11b^hi^, Gr1^hi^ staining and enumerated by taking into account the lavage volume recovered. The remaining lavage leukocytes were counted by hemocytometer and analyzed by Kwik-Diff staining of cytospins as an alternative method of identification. Both methods of quantification gave essentially the same results.

To induce peritonitis with LPS, mice were challenged *i.p.* with 250 ng LPS (Sigma) in 250 μl sterile saline, or were mock-treated with saline, and euthanized by CO_2_ asphyxiation after 3 h. Peritoneal lavages were performed and lavage leukocytes analyzed by flow cytometry and Kwik-Diff staining of cytospins as described above.

### Neutrophil purification

Mature primary neutrophils were freshly isolated each day by Percoll^PLUS^ gradient at 4°C from mouse bone marrow using endotoxin-free reagents throughout, essentially as previously described (35). Mouse bone-marrow was flushed from femurs, tibias and pelvic bones with ice-cold Hank’s Balanced Salt Solution without Ca^2+^ or Mg^2+^ (HBSS^–^, Sigma H6648) supplemented with 15 mM HEPES, pH 7.4 (RT) (Sigma, H3784) and 0.25% fatty acid-free (FAF) BSA (Sigma, A8806) (HBSS^–++^), triturated and filtered through a 40 μm cell strainer. 58% isotonic Percoll^PLUS^ (GE Healthcare, 17544501) in HBSS^–++^ was underlayed and samples spun at 1620 × g without brake for 30 min at 4°C. For mass spectrometric analysis of the neutrophil proteome, this gradient step was repeated once, by collecting the lower 5 ml and subjecting it to another Percoll^PLUS^ gradient. After gradient centrifugation, the lower 5 ml were resuspended in 40 ml HBSS^–++^ and centrifuged at 326 × g for 10 min at 4°C. Erythrocytes were lysed in Geye’s solution for 3 min at RT. 10 volumes of ice-cold HBSS^–++^ were added and cells sedimented again. Neutrophils were resuspended in ice-cold DPBS^++++^ and kept on ice while aliquots were counted by hemocytometer and purity assessed by Kwik-Diff of cytospins. Preparations were >90% pure, except for mass spectrometric analysis where purity was 95-98% due to the use of a second density gradient. Neutrophils were sedimented again and resuspended in the buffer appropriate for the subsequent assay.

### Killing of bacteria *in vitro*

The ability of isolated neutrophils to kill *E. coli* was measured essentially as described (35). *E. coli* DH5α bacteria (New England Biolabs, 2527) were grown in LB to logarithmic phase on the day of the experiment. Bacteria were enumerated by OD_600_, sedimented, resuspended in ice-cold DPBS^++++^ at 2.5 x 10^9^/ml, opsonized with 50% mouse serum for 15 min at 37°C, washed in ice-cold DPBS^++++^, resuspended in DPBS^++++^ at 2.5 x 10^8^/ml and kept on ice. Purified neutrophils were resuspended in DPBS^++++^ at 2.5 x 10^7^/ml and kept on ice. Neutrophils were then either primed with 1 μg/ml LPS for 90 min at 37°C, or kept on ice for 45 min and then primed with 20 ng/ml TNFα and 50 ng/ml GM-CSF for 45 min at 37°C, or were heat-killed at 55°C for 45 min as a negative control. Opsonized bacteria and heat-killed neutrophils were prewarmed to 37°C for 3 min prior to the assay. One volume of opsonized bacteria was added to 4 volumes of primed or dead neutrophils at a ratio of 2.5 bacteria per neutrophil, and samples were incubated at 37°C. Aliquots were removed after 30 and 120 min, diluted 1:20 in ice-cold LB, 0.05% saponin, and incubated on ice for 10 min with frequent vortexing. Serial dilutions were made in LB, and samples were plated onto LB agar and incubated overnight at 37°C. Bacterial CFU in samples with live neutrophils were expressed as % of samples with heat-killed neutrophils.

### ROS

ROS production was measured by luminol chemiluminescence assay in a Berthold MicroLumat Plus luminometer (Berthold Technologies), essentially as previously described (37). Purified bone-marrow derived neutrophils were resuspended at 5 × 10^6^ cells/ml in ice-cold DPBS^++++^ and primed with 5 ng/ml TNFα, 100 ng/ml GM-CSF, or with TNFα or GM-CSF alone where indicated, for 45 min at 37°C, or with 1 μg/ml LPS (E. coli LPS, Sigma, L3024) for 90 min at 37°C, with occasional flicking to prevent settling, or they were mock-primed in DPBS^++++^ under the same conditions. Unprimed neutrophils were kept on ice and prewarmed to 37°C for 3 min prior to the assay. Stimuli (fMLP, C5a, PMA) were prepared as 2.5x stocks in DPBS^++++^. Prior to the assay, an equal volume of prewarmed Detect buffer (DPBS^++++^ containing 16 units/ml horseradish peroxidase (HRP, Sigma, P8375) and 120 µM luminol (Sigma-Aldrich, 123072)) was added to the prewarmed (primed, mock-primed or unprimed) neutrophils. The neutrophils/Detect mix was incubated for 3 min at 37°C, before 150 μl/well were dispensed into a prewarmed 96-well luminometer plate. 100 μl of prewarmed 2.5x stimulus in DPBS^++++^, or DPBS^++++^ control, was added either by automatic injection port (fMLP, C5a) or manually (PMA), and real-time ROS production was recorded at 37°C. Final assay concentrations were 1.5×10^6^ neutrophils/ml and 3 μM fMLP, 25 nM C5a, or 500 nM PMA. ROS production was quantified by integrating the area under the curve (AUC) of the ROS response over 2 min for fMLP and C5a, or over 10 min for PMA.

### Phagocytosis

Phagocytosis of *E. coli* was measured as follows. *E. coli* DH5α were grown in LB to logarithmic phase on the day of the experiment, enumerated by OD_600_, sedimented, resuspended in ice-cold DPBS^++++^ at 2.5 x 10^9^/ml, and opsonized with IgG opsonising reagent (Molecular Probes, E2870) according to manufacturer’s instructions. Purified bone marrow-derived neutrophils were resuspended at 1x10^7^ cells/ml in DPBS^++++^ and primed with 1 μg/ml LPS, or mock-primed with DPBS^++++^, for 90 min at 37°C. 100 μl of neutrophils were allowed to adhere to glass coverslips in a 24-well plate for 15 min at 37°C, 5% CO_2_. 100 μl of opsonised *E. coli* were added at a ratio of 25 bacteria per neutrophil, and samples were incubated for a further 120 min. Samples were fixed in 4% paraformaldehyde, PBS for 15 min at RT, washed twice in PBS, permeabilized in 0.1% Triton X-100/PBS for 10 min at RT and washed twice in PBS. Samples were stained with anti-rabbit IgG AF568 (Thermo Fisher Scientific, A11036, 1:400), FITC-Gr1 antibody (Thermo Fisher Scientific, RM3001, 1:500) and Hoechst 33342 DNA dye (Thermo Fisher Scientific 62294, 1:400), in PBS containing Fc block (1:100), for 40 min at RT, followed by three washes in PBS. Samples were mounted in ProLong Gold Antifade mountant (Life Technologies), imaged using a Zeiss Axio Imager D2 widefield system, and images were analyzed using ImageJ.

Phagocytosis of zymosan yeast particles was assayed essentially as described (35). Zymosan A particles (Molecular Probes, Z2894) were opsonised using IgG opsonising reagent (Molecular Probes, Z2850) according to the manufacturer’s instructions and stored in HBSS^++++^ at 4°C. Purified bone marrow-derived neutrophils were resuspended at 1 x10^7^ cells/ml in DPBS^++++^ and primed with 1 μg/ml LPS or mock-primed with DPBS^++++^ for 90 min at 37°C. 100 μl neutrophils were then allowed to adhere to glass coverslips in a 24-well plate for 15 min at 37°C, 5% CO_2_ before 100 μl opsonised zymosan particles were added at a ratio of 5 particles per neutrophil and samples incubated for a further 30 min at 37°C, 5% CO_2_. Samples were fixed, permeabilized, stained, imaged, and images analyzed as described here above.

### Degranulation

Degranulation of myeloperoxidase (MPO) from azurophil granules in response to stimulation with *E. coli* was measured as follows. *E. coli* DH5α were grown in LB to logarithmic phase on the day of the experiment, enumerated by OD_600_, sedimented, and resuspended in ice-cold DPBS^++++^ at 2.5 x 10^9^/ml. Purified bone marrow-derived neutrophils were resuspended at 1.82 × 10^7^/ml in DPBS^++++^, and 1 x 10^6^ cells were stimulated with *E. coli* (at a ratio of 12.5 bacteria per neutrophil), or were mock-stimulated with DBPS^++++^. Either immediately (0’ control), or after 3 h, the cells were pelleted at 326 × g for 10 min at 4°C, and the supernatant was removed and centrifuged at 10,000 × g for 1 min. Cell pellets were washed in DPBS^++++^. The cleared supernatant and washed cell pellets were boiled in SDS-PAGE sample buffer for 5 min, and samples were analyzed by SDS-PAGE and western blotting with MPO antibody (R&D Systems, AF3667, 1:3000), followed by ImageJ analysis. The percentage of secreted MPO was calculated as % of the total MPO in the 0’ cell pellet.

Gelatinase (MMP9) degranulation was measured by in-gel zymography essentially as described (38). Purified bone marrow-derived neutrophils were resuspended at 5 × 10^6^/ml in DPBS^++++^ and primed with 1 μg/ml LPS for 90 min at 37°C, or were mock-primed in DPBS^++++^, or were kept unprimed on ice. Neutrophils (80 μl/well) were pipetted into a 96-well plate (Nunc; pre-blocked with 10% heat-inactivated fetal bovine serum, FBS) containing 20 μl 5x fMLP and cytochalasin B in DPBS^++++^, or DPBS^++++^ alone. Final concentrations were up to 1 μM fMLP and 10 μM cytochalasin B. Cells were incubated for 30 min at 37°C in 5% CO_2_, and then centrifuged at 300 × g for 10 min at 4°C. 40 μl of the conditioned supernatant was mixed with 20 μl 3x non-reducing SDS-PAGE sample buffer (160 mM Tris, pH 6.8, 8% SDS, 50% glycerol, bromophenol blue) at RT. 10 μl aliquots were separated by SDS-PAGE gel containing 0.067% gelatine B. Gels were equilibrated in 2.5% Triton X-100 for 30 min and in developing buffer (50 mM Tris, pH 7.5, 200 mM NaCl, 5 mM CaCl_2_, 0.02% Triton X-100) overnight at RT, allowing the gelatinase to digest the gelatine in the gel. Gels were coomassie-stained and gelatinase activity analyzed by densitometry using ImageJ software.

### Release of NETs

Release of NETs in response to stimulation with *E. coli* was measured exactly as described above for the degranulation of MPO (same samples used for both assays), except that samples were western blotted for citrullinated histone H3 (CitH3 antibody, Abcam ab5103, 1:4000). In addition, DNA in the cell pellets and supernatant was quantified by spectrometry.

NET production in response to stimulation with *Staphylococcus aureus* (*S. aureus, Wood 46*) was done essentially as described (35). *S. aureus* were subcultured in LB to logarithmic growth at 37°C, sedimented for 2 min at 12,000 x g, washed in Dulbecco’s Modified Eagle Medium with Ca^2+^, Mg^2+^ and 4.5 g/l glucose (DMEM^+++^, Thermo Fisher Scientific, 31053) supplemented with 10 mM Hepes, pH 7.4 (DMEM^+++^/Hepes), and opsonized with 10% mouse serum for 30 min at 37°C. *S. aureus* were washed twice in DMEM^+++^/Hepes following opsonization and resuspended at 5x10^7^ bacteria/ml in DMEM^+++^/Hepes. Purified bone marrow-derived neutrophils were resuspended at 4x10^5^ cells/ml in DMEM^+++^/Hepes containing 10% heat-inactivated FBS and primed with 1 μg/ml LPS or mock-primed for 90 min at 37°C. 250μl of cells were then seeded into each well of an 8-well chamber slide (μ-slide 8 well, 80826, ibidi) and allowed to adhere for 30 min at 37°C, 5% CO_2_. Cells were treated with opsonised *S. aureus* at a ratio of 10 bacteria per neutrophil or mocked-treated with DMEM^+++^/Hepes/FBS for the indicated periods of time. 15 min before the end of the incubation period, the non-cell permeable DNA dye Sytox Green (Thermo Fisher Scientific, S7020, 0.1μM) and the cell permeable DNA dye Hoechst 33342 (Thermo Fisher Scientific, 62294) were added to cells and samples were live-imaged using a Nikon Eclipse Ti-E widefield system. Images were analyzed by ImageJ software, using phase contrast and DAPI stain to determine the total cell number and Sytox Green signal to enumerate cells with NETs.

### Chemotaxis

Transwell chemotaxis assays were done essentially as described (37) using 3 μM-pore polycarbonate filters (Millipore, Millicell-PC, PITP01250) in ultra-low cluster 24-well tissue culture plates (Costar, 3473). Bone marrow was flushed into HBSS with Ca^2+^ and Mg^2+^ (Sigma, H8264), supplemented with 0.25% fatty acid-free BSA, and 15 mM Hepes, pH 7.5 at 37°C, all endotoxin-free (HBSS^++++^), triturated, filtered through a 40 μm nylon cell strainer, counted by hemocytometer and adjusted to 5 x 10^6^/ml. Cells were primed with 1 μg/ml LPS for 90 min, or with 20 ng/ml murine TNFα and 50 ng/ml GM-CSF for 45 min, or were mock-primed in HBSS^++++^ for the same periods of time at 37°C. Cells were pipetted into transwell filters (400 μl/filter) in a 24-well plate containing HBSS^++++^ (600 μl/well) in the presence or absence of 3 nM C5a or 1 μM fMLP, and were incubated for 40 min at 37°C. Cells remaining in the transwell were removed and replaced with 400 μl ice-cold HBSS^–++^ containing 3 mM EDTA. 60 µl HBSS^–++^ containing 30 mM EDTA was added to the bottom well and plates were incubated on iced metal trays for 15 min to detach cells. Transmigrated cells were collected from the bottom well, and in parallel to control cells that had not undergone chemotaxis, were centrifuged at 10,000 xg for 30 s and resuspended in ice-cold HBSS^–++^. Cells were stained with FITC-Gr1 (clone RB6-8C5, BD Pharmingen, 553126, 1:800) and AF647-Cd11b (clone M1/70, BD Pharmingen, 557686, 1:800) antibodies in HBSS^–++^ containing 1% Fc block and were analyzed using an LSR2 flow cytometer alongside Spherotech ACBP-50-10 standard beads. Neutrophils were identified by their Gr1^hi^/Cd11b^hi^ staining. Transmigrated neutrophils were compared to total neutrophils in control samples.

For Ibidi chamber chemotaxis assays, 6-channel ibidi slides (µ-slide VI 0.4, ibidi 80601) were coated with 20 μg/ml fibronectin-like poly-Arg-Gly-Asp peptide (pRGD, Sigma, F5022) or with 1 mg/ml fibrinogen in DPBS^–^ for 1 h at RT and washed 3 times in HBSS^++++^, and the wells and central chamber were filled with HBSS^++++^. Isolated neutrophils were resuspended at 2 x 10^7^/ml in HBSS ^–++^ and kept on ice until use. Cells were primed by adding 5 µl to 90 µl of HBSS^++++^ supplemented with 1g/l glucose and containing 20 ng/ml TNF-alpha and 50 ng/ml GMCSF for 45 min at 37°C, or were mock-primed. Buffer was removed from both wells of the ibidi slide before adding the cells, leaving the central chamber full. 45 µl of primed or mock-primed cells were added into one well and 45 µl of liquid removed from the other to draw the cells through into the central chamber. The slide was inserted into the microscope housing, and cells were allowed to adhere for 20 min at 37°C. 5 μl of HBSS^–++^ containing 10 µM fMLP, or other concentrations where indicated, and 5 x 10^6^ carboxyl polystyrene beads (Bangs Laboratories, PC06N, 6.9 μm) was then added to one well and 5 µl of buffer removed from the other to create an fMLP gradient, or HBSS^–++^ alone was used for mock-stimulation. The beads were used to locate the steepest part of the gradient where imaging was done. Neutrophils were live-imaged for 20 min every 10 s in a prewarmed Olympus CellR microscope using the 20x objective. Cells were tracked manually using the chemotaxis and migration plugin of ImageJ software to quantify various parameters of cell speed and directionality as indicated. Responders were defined as cells that migrated at least their own cell-length over the 20 min observation period.

### Cell surface receptor levels

Bone marrow cells were flushed with ice-cold HBSS^–++^, filtered through 40 μm cell strainers, counted, pelleted at 326 × g for 10 min at 4°C, and resuspended in ice-cold DPBS^++++^ at 4 × 10^7^ cells/ml. 125 μl of cells were either kept on ice, or were primed with 20 ng/ml TNFα and 50 ng/ml GM-CSF, or with 1 μg/ml LPS, for 45 min at 37°C. Cells were sedimented at 10,000 × g for 30 s at 4°C, resuspended in Fc block (BD Biosciences, clone 2.4G2, 1:1000; for Mac1 and L-selectin) or in DPBS^++++^ (for FcγRIII), and incubated on ice for 15 min. Cells were sedimented at 10,000 × g for 30 s, resuspended in ice-cold DPBS^++++^ containing fixable viability dye (eBioscience, eFluor™ 780, 1:1000), antibodies for neutrophil markers Ly6G (Ly6G-BV510, BioLegend, clone 1A8, 1:500) and Mac1 (CD11b-AF647, BD Bioscience Clone M1/70, 1:1000), and PE-labelled antibodies for FcγRIII (CD16, BioLegend, clone S17014E, 1:100) or L-selectin (BD Biosciences clone MEL-14, 1:100), and were incubated on ice for 30 min. Cells were washed in ice-cold HBSS^–++^, 1 mM EDTA, resuspended in 300 μl ice-cold HBSS^–++^, 1 mM EDTA, and kept on ice. Flow cytometry was performed using a BioRad ZE5 flow cytometer, recording 20,000 neutrophils per sample. Neutrophils were identified by Ly6G^hi^, CD11b^hi^ staining, and the mean fluorescence intensity (mfi) of receptor levels on the neutrophil surface was quantitated using FlowJo.

### Neutrophil proteomics

Neutrophils were isolated to 95-98% purity as described above with two consecutive Percoll^PLUS^ gradients from the pelvic bone-marrow of 8 young (8 weeks) and 8 old (24 months) mice, in 4 independent experiments with 2 young and 2 old mice per experiment. Separately for each mouse, cells were resuspended at 2 x 10^7^/ml in DPBS^++++^, treated with the cell-permeable serine protease inhibitor diisopropyl-fluorophosphate (DFP, 7 mM, Sigma, D0879) for 10 min at RT, washed twice in DPBS^++++^, sedimented for 30 s at 10,000 x g, and the cell pellets snap-frozen in liquid nitrogen and stored at -80°C prior to further processing. Proteins were solubilized and reduced in 6 M guanidine-HCl, 100 mM Tris, 10 mM DTT for 1 h at 50°C, then cooled and alkylated with 50 mM iodo-acetamide for 30 min in the dark. The alkylated proteins were precipitated with 4 volumes of acetone for 16 h at -20°C, then solubilized in 100 mM triethyl-ammonium bicarbonate, 6 M guanidine hydrochloride, and sequentially digested with Lys-C and trypsin (Promega) for 16 h at 30°C. The resulting peptides were labelled with tandem mass-tags using two sets of 8-plex reagents (Thermo Scientific). The two combined sets of 8 samples were each separated into 60 fractions by high-pH reverse phase HPLC. The major peptide-containing fractions were analyzed by LC-MS on an Orbitrap Fusion Lumos mass spectrometer, using an SPS-MS3 scan sequence. Proteins were identified from the mass spectrometry data by searching the Uniprot mouse proteome database using Mascott software (Matrix Science), and relative abundances of the identified proteins were determined using Proteome Discoverer software (Thermo Scientific). This identified 8001 proteins in total. Non-mouse derived proteins (e.g. human keratins, bovine serum albumin) were removed from the list, which left 7945. Only proteins detected in at least 4 old and 4 young mice were analyzed further, which left 7338. Statistical analysis of the relative protein abundances was done using R software to calculate differences between young and old by two-sided t-tests on log-transformed data, with Benjamini-Hochberg false-discovery rate correction for multiple comparisons of all 7338 quantified proteins. Proteins were assigned to one or more categories of protein class or signaling pathway using a combination of PANTHER pathway analysis (www.pantherdb.org) and manual curation.

### LPS/TLR4 pathway activity

Purified bone marrow-derived neutrophils were resuspended at 1x10^7^ cells/ml in DPBS^++++^, primed with 1 μg/ml LPS or mock-primed with DPBS^++++^ for 90 min at 37°C, and stimulated with pre-warmed fMLP (1 μM final) for one min. The reaction was stopped by addition of excess ice-cold PBS, followed by centrifugation at 12,000 x g for 10 s at 4°C. The cell pellet was lysed in lysis buffer (20 mM Tris [pH 7.5, 4°C], 150 mM NaCl, 1 mM EDTA, 1 mM EGTA, 1% Triton X100, 2.5 mM Na pyrophosphate, 1 mM β-glycerol-phosphate, 1 mM Na orthovanadate, 4 mM DTT, 0.1 mM PMSF, and 10 μg/ml of each aprotinin, pepstatin, antipain and leupeptin) for 10 min on ice with frequent vortexing. Debris was sedimented at 12,000 x g for 10 min at 4°C, and the lysates were boiled in SDS-PAGE sample buffer and subjected to SDS-PAGE and western blotting using antibodies for phosphorylated p38 Mapk, p42/44 Erk and Akt. Blots were stripped and reprobed with antibodies for total p38 Mapk, p42/44 Erk and Akt. Antibodies are listed in Materials. Blots were quantitated by ImageJ densitometry, and phospho-protein signals normalized to total-protein for each sample.

### Western blots

To evaluate protein expression in neutrophils, total lysates were prepared from mature neutrophils isolated from bone marrow as described above, were treated with 7 mM DFP for 10 min at RT, and washed in DPBS^++++^. Cells were sedimented or 30 s at 10.000 xg, resuspended in boiling 1.3 x SDS-PAGE sample buffer, and boiled for 5 min with repeated trituration through a 25 x G needle. Proteins were separated by SDS-PAGE, transferred onto PVDF, blocked and probed with the appropriate antibodies, and detected using ECL or ECL Prime (GE Healthcare). Where required, membranes were stripped in 25 mM glycine, pH 2.0, 1% SDS for 5 min at RT and reprobed with different antibodies. Protein loading was assessed by coommassie staining. Densitometric analysis of western blots was done manually using the wand tool of ImageJ software, essentially as described in ‘www.sybil-fp7.eu/node/95‘, except that bands of interest were delineated on the ImageJ plots using two vertical lines through the whole gel, and background was subtracted from the equivalent areas of an empty lane, to avoid bias.

### Rac activity

Rac activity was assessed by Pak-CRIB pull down, essentially as described (18). GST-Pak-CRIB bait was purified from bacterial culture as described (39) and stored in GST-FISH buffer (10% glycerol, 50 mM Tris pH 7.4, 100 mM NaCl, 1% NP-40, 2 mM MgCl_2_, 2 mM DTT, 100 µM PMSF, and 10 µg/ml each of leupeptin, pepstatin A, aprotinin and antipain) at 4°C for up to one week. Purified bone marrow-derived neutrophils were resuspended at 1 × 10^7^/ml in DPBS^++++^ and pre-warmed for 3 min at 37°C. 200 μl aliquots were stimulated with 10 x fMLP (Sigma, F3506) at the indicated concentrations in DPBS^++++^ for 10 s, or were mock-stimulated. The reaction was stopped by the addition of 1 ml of ice-cold GST-FISH buffer containing 1.2% NP-40 (to give a final concentration of 1% NP-40), and cells were lysed by incubation on ice for 2 min with frequent vortexing. Samples were centrifuged at 12,000 x g for 3 min at 2°C to sediment debris, the supernatant transferred into fresh precooled tubes, and 2% kept as a total lysate control. The remaining sample was incubated with GST-Pak-CRIB beads by end-over-end rotation for 15 min on ice. Samples were washed 5 times in GST-FISH buffer before adding boiling 1.3x SDS-PAGE sample buffer and boiling the samples for 5 min. To process total lysate samples, boiling 4x SDS-PAGE sample buffer was added to final 1.3x, and samples boiled for 5 min. GTP-Rac and total Rac were quantified by western blotting with Rac1 and Rac2 antibodies and densitometry using ImageJ.

### PIP_3_ and PIP_2_ measurements

PIP_3_ production by class I PI3K, and the amount of PIP_2_ in the cell, were measured directly by lipid mass spectrometry (40). Isolated bone-marrow derived neutrophils were resuspended in DPBS^++++^ at 7.4 x 10^6^ per ml and primed with 1 μg/ml LPS for 90 min, or with 20 ng/ml murine TNFα and 50 ng/ml GM-CSF for 45 min, or mock-primed for the same periods of time in DPBS^++++^ at 37°C. 1 x 10^6^ cells were then stimulated for 10 s with 3 µM fMLP or 25 nM C5a, or were mock-stimulated in DPBS^++++^. The reaction was stopped by the addition of 750 µl CHCl_3_:MeOH (1:2). Synthetic deuterated stearoyl/arachidonoyl PIP_3_ (10 ng) and PI(4,5)P_2_ (100 ng) were added to each sample as internal standards. Lipids were extracted as previously described (41) and analyzed by high performance liquid chromatography-mass spectrometry (HPLC-MS) (40). The stearoyl/arachidonoyl species of PIP_2_ and PIP_3_ were quantitated, as they are the most abundant species of these lipids in neutrophils (40). For quantification, the area of the PIP_2_ and PIP_3_ peaks was integrated and compared to the relevant internal standard.

### Data collection and statistical analysis

Sample size was determined using power calculations to yield 80% power, based on results of pilot experiments and on previously published data as referenced. Experiments were performed at least three times except where indicated. Sample size and numbers of independent experiments are detailed in Figure Legends. Within the parameters of group size and age, mice were selected for cohorts at random by the staff of the Small Animal Unit. Image analysis was performed in a blinded manner. Excel 2016 and GraphPad Prism 8.0 were used for tabulation, statistical analysis and plotting graphs, except for proteome analysis where R software was used. Data were tested for normality of distribution using Shapiro-Wilk test to determine if parametric or non-parametric statistical analysis was required. Where warranted by variance between groups, data were log-transformed prior to statistical analysis. Statistical outliers were identified using Tukey’s test and removed from datasets. Other samples were only excluded when there was a known technical problem affecting the analysis. For the analysis of proteomics data, comparisons between the age groups were made by two-tailed unpaired Student’s t-test. For all other data, two-way or three-way ANOVA was used to test for effects of interventions. Group sizes (n) are listed in figure legends. Effect size and variance are reported as group mean ± standard error. P-values reported for proteomics data are from t-tests with Benjamini-Hochberg false discovery rate correction for multiple comparisons, p-values reported for other data are from multiplicity-adjusted Sidak’s *post-hoc* comparisons. The threshold for statistical significance was set at p<0.05.

## Results

### Old mice have impaired immunity against *E. coli* infection and altered neutrophil recruitment during septic peritonitis

**Figure 1:**
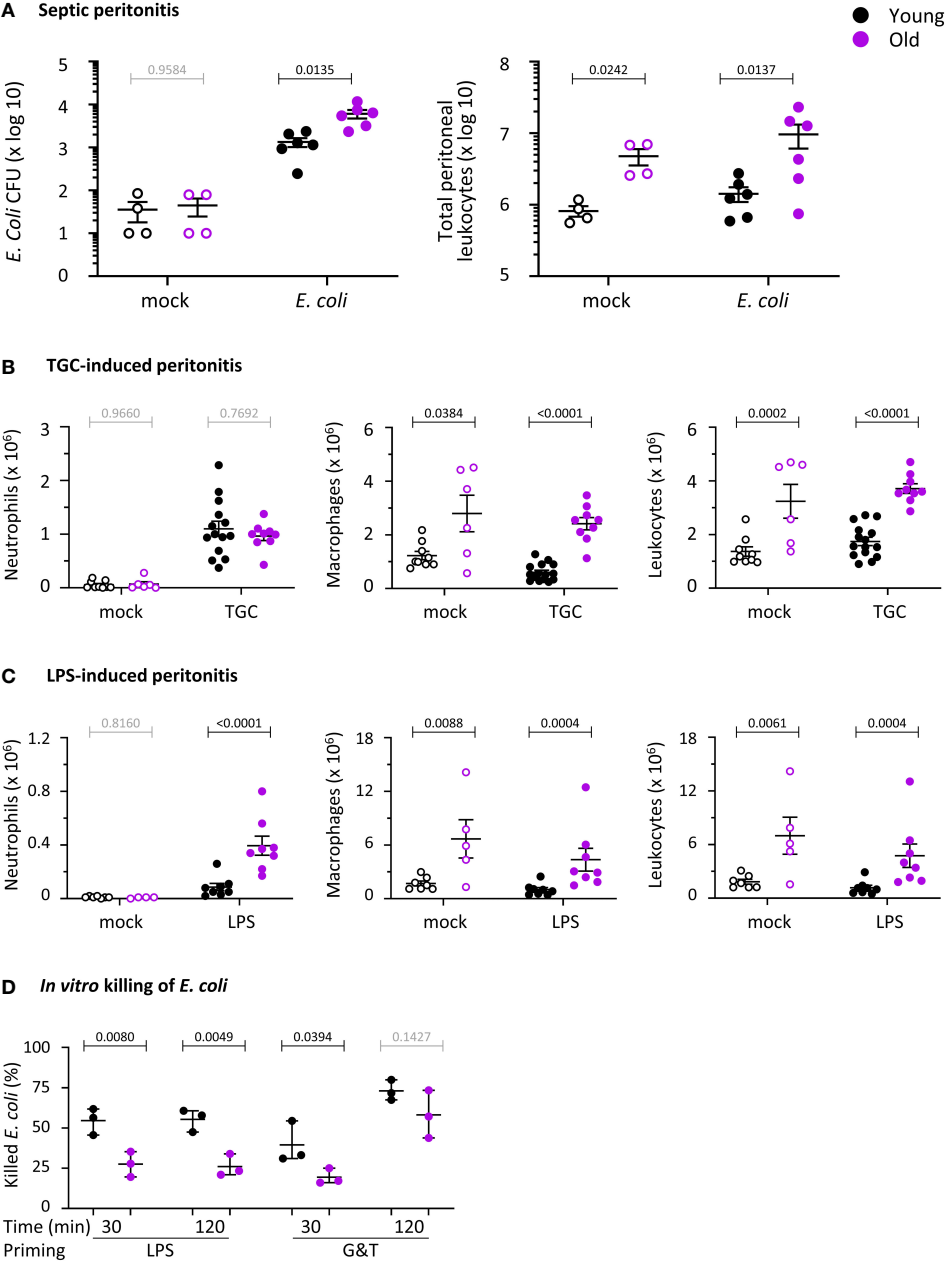
Hornigold K et al. Old mice have impaired immunity against *E. coli* infection and altered neutrophil recruitment, and the ability of their neutrophils to kill *E. coli* is reduced. **(A)** *E. coli*-induced septic peritonitis. Young (8-10 weeks, black symbols) and old (24 months, purple symbols) mice were infected *i.p.* with 1x10^4^ pathogenic *E. coli* O18:K1 (filled symbols), or were mock-infected (open symbols), and culled humanely 3 h later. Peritoneal lavages were performed and assessed for bacterial burden by bacterial culture and quantification of CFU. Lavage neutrophils and total leukocytes were quantified by microscopy. Data are mean ± SEM of mice pooled from 3 independent experiments, with 1-2 mock-infected and 1-3 infected mice/age/experiment. **(B)** Aseptic peritonitis. Young and old mice were challenged *i.p.* with 0.25 ml sterile 3% thioglycollate (TGC, filled symbols), or were mock-treated (open symbols), and culled 3 h later. Peritoneal lavages were assessed for numbers of peritoneal neutrophils, macrophages and total leukocytes by hemocytometer and microscopy of cytospins. Data are mean ± SEM, pooled from 5 independent experiments, with 1-3 mock-treated and 1-4 TGC-treated mice/age/experiment. **(C)** LPS-induced septic peritonitis. Young and old mice were challenged *i.p.* with 250 ng LPS in 0.25 ml saline (filled symbols), or were mock-treated with saline (open symbols), and culled 3 h later. Peritoneal lavages were assessed as in **(B)**. Data are mean ± SEM, pooled from 3 independent experiments, with 1-2 mock-treated and 2-3 LPS-treated mice/age/experiment; dots represent individual mice. Statistics in **(A-C)** are two-way ANOVA with Sidak’s multiple comparisons test on log-transformed raw data; black p-values are significant, grey p-values non-significant. **(D)** *In vitro* killing of *E. coli*. Neutrophils purified from young (8-10 weeks, black symbols) and old (24 months, purple symbols) mice were primed with 1 μg/ml LPS or with 20 ng/ml TNFα and 50 ng/ml GM-CSF, as indicated, or were heat-killed as a negative control, and were incubated with serum-opsonised *E. coli* DH5α (ratio of 2.5 bacteria per neutrophil) at 37°C for 30 or 120 min. Bacterial killing by neutrophils was quantified by comparing CFU between samples containing live and heat-killed neutrophils. Data are mean ± SEM of 3 independent experiments; each dot represents the mean of one experiment. Statistics are three-way ANOVA with Sidak’s multiple comparisons test; black p-values are significant, grey p-values non-significant.

In humans, the incidence of bacterial peritonitis increases with age, associated with high mortality rates from sepsis (4). To investigate if antibacterial immunity wanes similarly in aging mice, we compared young (8-10 week-old) and old (24 months-old) mice. These mouse ages are equivalent to the human ages of 20 and 80 years, respectively (42). We tested the ability of the mice to clear pathogenic *E. coli* strain O18:K1 in an acute model of septic peritonitis. This model tests the early innate response (after 3 h) to an infection that would prove fatal to 75% of the mice within 36 h if left to progress (34). At the acute stage tested here, only neutrophils are recruited, to combat the infection together with resident peritoneal macrophages (35). Old mice showed a 4.6-fold reduction in their ability to clear the infection, despite constitutively having 6-fold more peritoneal leukocytes than young mice and double the leukocyte recruitment in response to the infection (**Figure 1A**). Hence, as for elderly humans, the innate immunity of mice to septic peritonitis decreases with age. This result reinforces previous reports showing reduced innate immunity of old mice to other types of bacterial and fungal infections (15–17).

To investigate neutrophil recruitment, we compared two peritonitis models, thioglycollate (TGC)-induced aseptic peritonitis and septic LPS-induced peritonitis. The latter recapitulates in part the *E.coli* infection, as LPS is the main endotoxin derived from gram-negative bacteria like *E.coli*, driving the inflammation that leads to sepsis. Neutrophil recruitment into the peritoneum was normal during aseptic peritonitis (**Figure 1B**) but increased in response to LPS (**Figure 1C**). Furthermore, peritoneal macrophage numbers were constitutively elevated in old mice, accounting for the high number of total peritoneal leukocytes (**Figures 1B, C**). Altogether, defective neutrophil recruitment and peritoneal macrophage numbers do not seem to underlie the impaired antibacterial immunity of old mice, suggesting functional defects in one or more of these cell lineages.

We tested the effector responses of isolated neutrophils from young and old mice. We chose to purify mature neutrophils from the bone-marrow, where a pool of mature cells is always stored for rapid release into the circulation in case of inflammation or infection. We reasoned that using these cells instead of peripheral neutrophils would minimize confounding effects from the neutrophil aging which occurs in the circulation.

### Killing of *E. coli* is impaired in neutrophils from old mice

Following from the *in vivo* infection experiments, we tested the capacity isolated neutrophils from young and old mice to kill *E.coli*. We primed neutrophils before the assay, either with LPS or with GM-CSF and TNFα, in order to mimic inflammatory conditions. Priming sensitizes neutrophils to mount larger effector responses in response to stimuli, and is based on receptors stored on granules being upregulated to the neutrophil surface (43). Neutrophils from old mice had a reduced ability to kill bacteria, regardless of whether they were primed with LPS or with GM-CSF and TNFα. However, the defect in GM-CSF/TNFα-primed cells was overcome by prolonged incubation of neutrophils with the bacteria, whereas the defect in LPS-primed cells persisted (**Figure 1D**). Overall, the impaired clearance of *E.coli* infections *in vivo* in old age may stem from an age-dependent loss of killing capacity in neutrophils.

### LPS-primed ROS production is impaired in neutrophils from old mice

**Figure 2:**
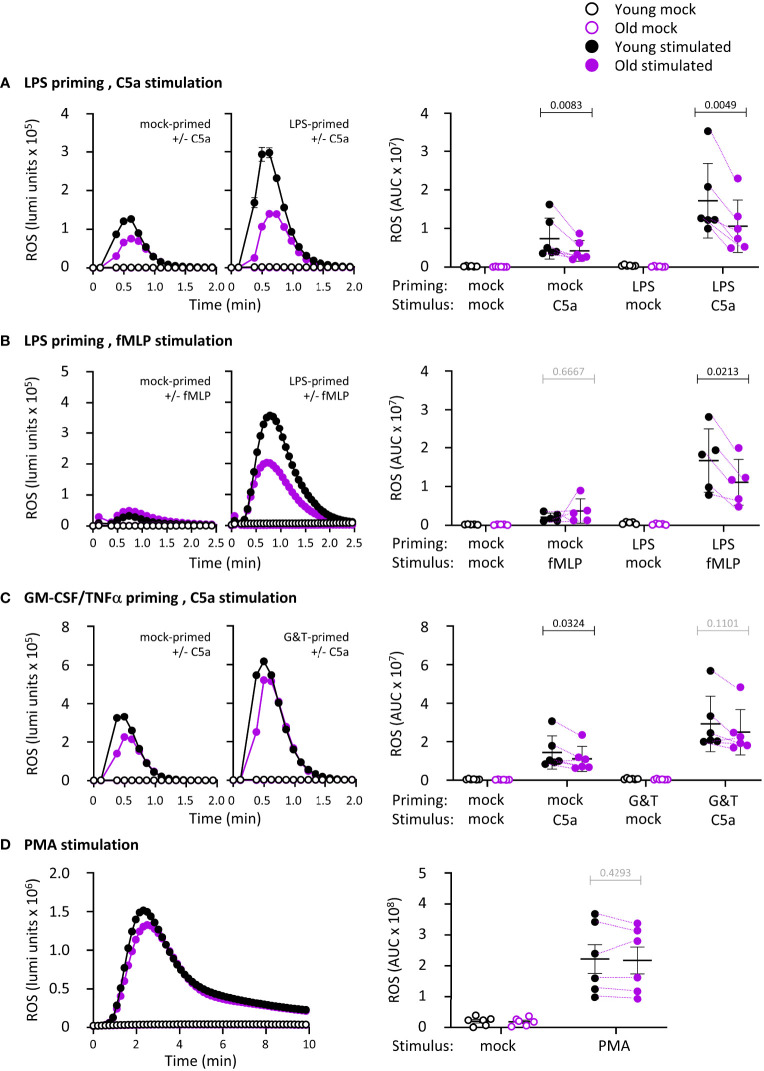
Hornigold K et al. LPS-primed ROS production is impaired in neutrophils from old mice. Isolated neutrophils from young (8-10 weeks, black symbols) and old (24 months, purple symbols) mice were primed with **(A, B)** 1 μg/ml LPS for 90 min or with **(C)** 100 ng/ml GM-CSF and 5 ng/ml TNFα for 45 min, or were mock-primed for the same periods of time, or **(D)** kept unprimed, prior to stimulation with **(A-C)** 25 nM C5a, **(B)** 3 μM fMLP or **(D)** 500 nM PMA (closed symbols), or mock-stimulation (open symbols), as indicated. ROS production was measured by real-time chemiluminescence assay with luminol and HRP for extra- and intracellular ROS. Left-hand panels show representative luminometer traces from one experiment; right-hand panels show the quantification as AUC. Data are mean ± SEM of 5-6 independent experiments, as indicated; each dot is the mean AUC from one experiment. Matched data are indicated by purple lines for stimulated cells. Statistics are two-way ANOVA with Sidak’s multiple comparisons tests on log-transformed raw data, in A-C for chemoattractant-stimulated cells; black p-values are significant, grey p-values non-significant.

ROS production is an important effector response in neutrophil-mediated immunity, so we tested the ability of isolated neutrophils from young and old mice to produce ROS. We primed neutrophils with LPS or with GM-CSF and TNFα, and stimulated them with the GPCR ligands and chemoattractants C5a, a component of the complement cascade, or fMLP, a peptide derived from bacterial proteins. Priming alone did not elicit ROS production, and in the absence of priming, C5a and fMLP induced only limited responses, as expected (**Figures 2A-C**). These data show that bone-marrow derived mature neutrophils from old mice are not pre-activated through effects of ‘inflamm-aging’. In mock-primed cells, C5a-stimulated ROS was lower in old age than young, whereas fMLP-stimulated ROS was not, showing an impairment dependent on the GPCR pathway (**Figures 2A-C**). Pilot experiments using priming with GM-CSF and TNFα separately or combined showed additive effects on fMLP-stimulated ROS production (**Supplemental Figure 1**). Hence, we decided to combine GM-CSF/TNFα throughout the study and juxtapose them to LPS. LPS or GM-CSF/TNFα effectively primed the C5a- and fMLP-stimulated ROS production in young age, as expected (p <0.0001 in A and C, p=0.0002 in B). In old age, the LPS-primed response was reduced (**Figures 2A, B**), whereas the GM-CSF/TNFα-primed response was not (**Figure 2C**). Therefore, there is an age-related decline in GPCR-dependent ROS production, with a degree of selectivity for the LPS-primed response over GM-CSF/TNFα. We also tested receptor-independent ROS production by activating the NADPH oxidase with PMA. PMA-stimulated ROS production was equal in neutrophils from young and old mice, showing that the overall capacity of the NADPH oxidase complex is not affected by age (**Figure 2D**).

### Phagocytosis is impaired in neutrophils from old mice, in part through effects of LPS

**Figure 3:**
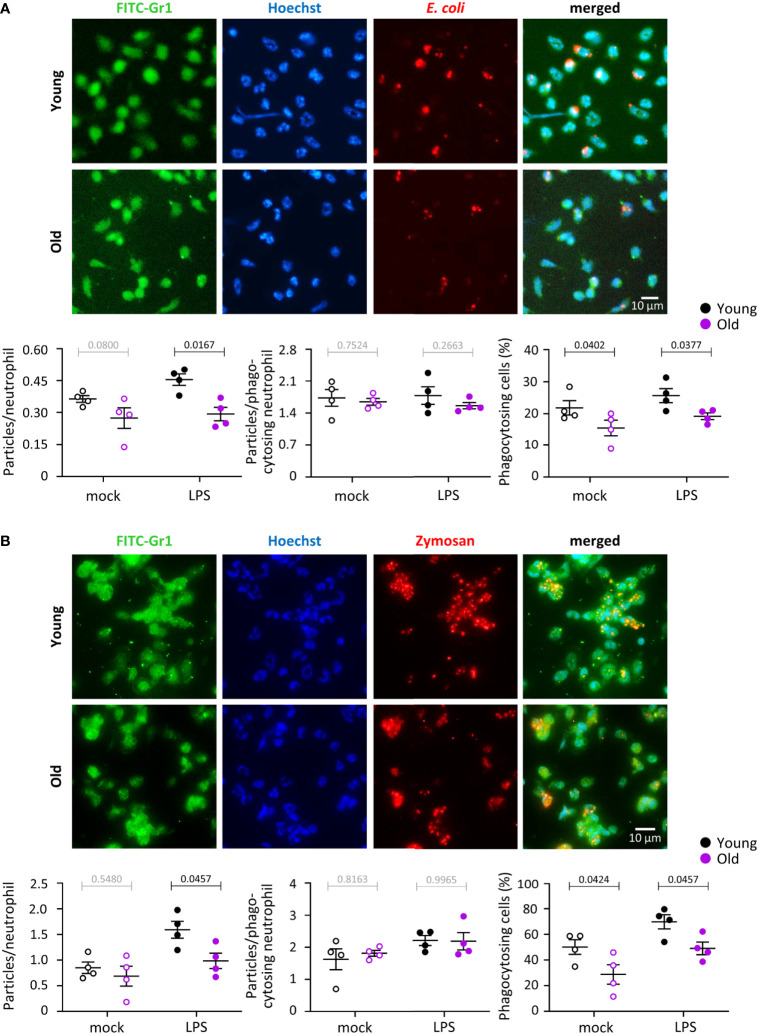
Hornigold K et al. Phagocytosis is impaired in neutrophils from old mice, in part through effects of LPS. Neutrophils from young (8-10 weeks, black symbols) and old (24 months, purple symbols) mice were primed with 1 μg/ml LPS for 90 min at 37°C (closed symbols), or were mock-primed (open symbols), and then plated onto glass coverslips for 15 min and stimulated either with IgG-opsonised *E. coli* (25 bacteria per neutrophil) for 120 min at 37°C **(A)**, or with IgG-opsonised zymosan yeast particles (5 particles per neutrophil) for 30 min at 37°C **(B)**. Cells were fixed, stained with FITC-Gr1 and AF568-IgG antibodies and Hoechst DNA dye, and assessed for the number of *E. coli* or zymosan particles within by widefield microscopy and ImageJ analysis. Representative images from one experiment are shown. Graphs in **(A)** and **(B)** show the quantification by ImageJ analysis for the number of particles per neutrophil overall (left panel), number of particles in neutrophils which had taken up at least one (middle panel), and proportion of phagocytosing neutrophils (right-hand panel). Data in **(A)** and **(B)** are mean ± SEM of 4 independent experiments; each dot represents the mean of one experiment. Statistics are two-way ANOVA with Sidak’s multiple comparisons tests; black p-values are significant, grey p-values non-significant.

We tested the capacity of neutrophils from young and old mice to phagocytose either IgG-opsonized *E. coli* or IgG-opsonized zymosan yeast particles in the presence or absence of LPS priming. In the context of the *E. coli* experiments, LPS priming mimics the inflammatory conditions encountered by neutrophils prior to contact with the bacteria, and for the zymosan experiments, it mimics co-infection of fungus (yeast) and gram-negative bacteria (LPS). The overall number of *E. coli* or zymosan particles taken up by neutrophils from old mice was reduced in LPS-primed cells but not in mock-primed cells (**Figures 3A, B**). The number of particles inside phagocytosing neutrophils was no different between the ages or priming conditions, but the proportion of neutrophils capable of phagocytosing was diminished in old age, both in LPS- and mock-primed cells, and for both stimuli (**Figures 3A, B**). Hence, phagocytosis is impaired in neutrophils from old mice in a partly LPS-dependent manner.

### Degranulation is impaired in neutrophils from old mice, in part through effects of LPS

**Figure 4:**
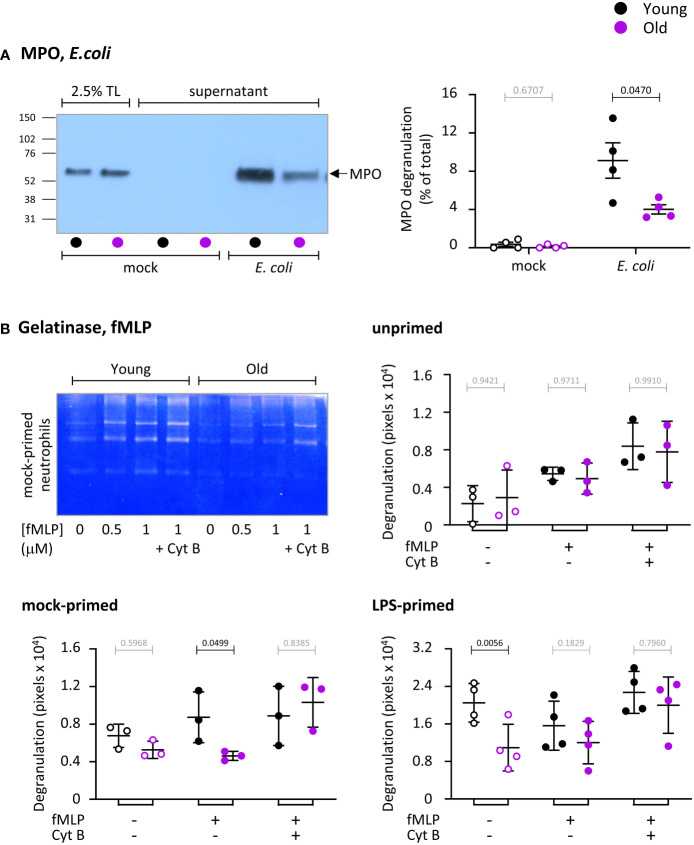
Hornigold K et al. Degranulation is impaired in neutrophils from old mice, in part through effects of LPS. **(A)** Degranulation of azurophil granules in response to *E.coli*. Neutrophils from young (8-10 weeks, black symbols) and old (24 months, purple symbols) mice were stimulated with *E. coli* (ratio of 12.5:1 bacteria/neutrophil; closed symbols), or were mock-stimulated with DBPS^++++^ (open symbols), for 3 h at 37°C. Cell pellets and cell supernatants were collected and western blotted for the azurophil granule marker MPO. Degranulation was quantitated by comparing secreted MPO to total MPO in the 0’ total lysate (TL) control. **(B)** Degranulation of gelatinase granules in response to fMLP. Neutrophils from young and old mice were kept unprimed on ice, or mock-primed at 37°C for 45 min, or primed with 1 μg/ml LPS for 90 min at 37°C, as indicated, and were then either mock-stimulated (open symbols), or stimulated with 1 μM fMLP, or with both 1 μM fMLP and 10 μM cytochalasin B (closed symbols) for 30 min at 37°C. Gelatinase activity released into the medium was analyzed by in-gel zymography. The representative coomassie-stained gel of mock-primed cells from one experiment shows the digestion of the gel by gelatinase as white areas. Quantification of gels was done by ImageJ densitometry. Data in **(A)** and **(B)** are mean ± SEM of 3-4 independent experiments, as indicated; each dot represents the mean of one experiment. Statistics are two-way ANOVA with Sidak’s multiple comparisons tests on log-transformed raw data; black p-values are significant, grey p-values non-significant.

We measured the ability of neutrophils from young and old mice to degranulate myeloperoxidase (MPO) from azurophil granules in response to *E. coli*, by western blotting cell pellets and cell supernatants for MPO. *E. coli* induced strong degranulation of MPO, which was higher in neutrophils from young mice than old, despite the total cellular content of MPO being the same between the ages (**Figure 4A**). Hence, degranulation of azurophil granules is impaired in old age.

We also measured the ability of neutrophils to degranulate gelatinase (MMP9) from gelatinase granules in response to fMLP, in the presence or absence of LPS priming, by using in-gel zymography to measure the secreted gelatinase activity (**Figure 4B**). Cytochalasin B, which depolymerizes the cortical actin ring of neutrophils thereby facilitating granule fusion with the plasma membrane, was used to test overall degranulation capacity. Stimulation of unprimed neutrophils with fMLP induced limited degranulation that was no different between the ages (**Figure 4B**). Mock-priming, which allows some vesicle trafficking, also resulted in limited degranulation, as expected (35). This was enhanced by fMLP stimulation in neutrophils from young mice but not old, showing there is a GPCR-dependent impairment (**Figure 4B**). LPS-priming induced robust degranulation in neutrophils from young but not old mice, showing there is also an LPS-dependent defect (**Figure 4B**). The LPS-primed response was not increased further by fMLP. The age-related defects in fMLP-stimulated mock-primed and LPS-primed cells were overcome by cytochalasin B treatment, demonstrating that LPS- and GPCR pathways were affected by age, rather than degranulation capacity overall. In contrast to LPS, GM-CSF/TNFα priming revealed no difference between the ages (data not shown). Hence, similar to ROS production and phagocytosis, neutrophils from old mice show both LPS-dependent and LPS-independent impairments in degranulation.

### Release of NETs in response to *S. aureus* but not *E. coli* is impaired in neutrophils from old mice, independently of LPS

**Figure 5:**
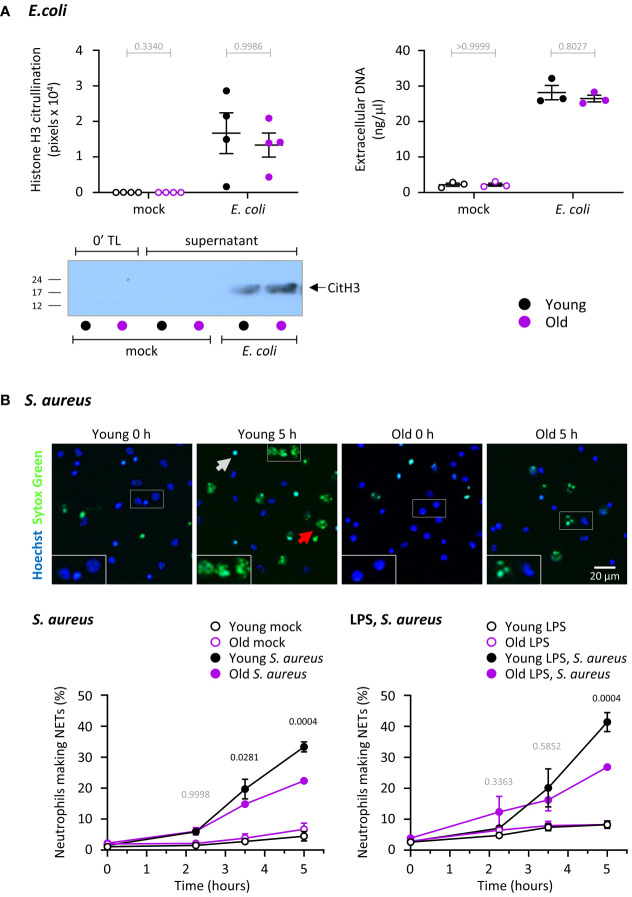
Hornigold K et al. NET release in response to *S. aureus* but not *E. coli* is impaired in neutrophils from old mice, independently of LPS. **(A)** NET release in response to *E. coli*. Release of DNA and citrullination of histone H3 were measured in the same samples as in **Figure 4A** to test NET release in neutrophils from young (8-10 weeks, black symbols) and old (24 months, purple symbols) mice in response to *E. coli*. DNA in cell pellets and cell supernatants was quantitated by spectrometry, and citrullinated H3 by western blotting. A representative blot from one experiment is shown. Data are mean ± SEM of 3-4 independent experiments, as indicated; each dot represents the mean of one experiment. Statistics (two-way ANOVA) revealed no differences between the ages (grey p-values). **(B)** NET release in response to *S. aureus*. Neutrophils from young and old mice were primed with 1 μg/ml LPS or mock-primed for 90 min at 37°C, seeded onto glass slides and allowed to adhere for 30 min at 37°C before stimulation with serum-opsonised *S. aureus* (10 bacteria per neutrophil; filled symbols), or mock stimulation (open symbols), for the indicated periods of time. Non-cell permeable Sytox Green and cell permeable Hoechst DNA dyes were added to samples 15 min before the end of incubation, and cells were live-imaged by widefield microscopy. **(A)** Representative images of mock-primed neutrophils from one experiment stimulated with *S. aureus* for the indicated lengths of time. The grey arrow indicates a dead cell, the red arrow a NET. **(B)** NETosis was quantify by ImageJ, using Sytox Green signal to identify NETs, and phase contrast and Hoechst signal to count total cells. Data are mean ± SEM of 7 independent experiments with mock-primed cells (left-hand panel) and 3 with LPS-primed cells (right-hand panel). Statistics are two-way ANOVA with Sidak’s multiple comparisons test. P-values denote significant differences between *S. aureus*-stimulated cells from young and old mice; black p-values are significant, grey p-values non-significant.

We measured the ability of neutrophils from young and old mice to release NETs in response to *E. coli*, using the same samples that were also tested for MPO degranulation (**Figure 4A**), by quantifying citrullination of histone H3 and the amount of DNA in cell pellets and cell supernatants. Unlike degranulation of MPO which was reduced in old age, extracellular release of DNA and citrullination of histone H3 were not affected by age under the same conditions (**Figure 5A**).

We also tested the ability of neutrophils to undergo *S. aureus*-induced NET release, in the presence or absence of LPS priming. *S. aureus* is a gram-positive bacterium, so in this context LPS priming was used to mimic co-infection with gram-negative bacteria. *S. aureus* induced robust NET formation which was stronger in neutrophils from young mice than old (**Figure 5B**). LPS priming alone hardly affected NET release, even when we used a species of LPS previously reported to induce NETs (data not shown) (44). LPS priming also had no major effect on *S. aureus*-induced NETs, meaning the response remained higher in cells from young than old mice (**Figure 5B**). Overall, neutrophils from old mice have a defect in NET release that depends on the type of bacteria but is independent of LPS. It should be noted that the defect was only observed after prolonged incubation (from 3 hours), which may explain why some previous studies failed to see the age-related impairment in NET release in mouse neutrophils.

### Migration is normal in neutrophils from old mice

**Figure 6:**
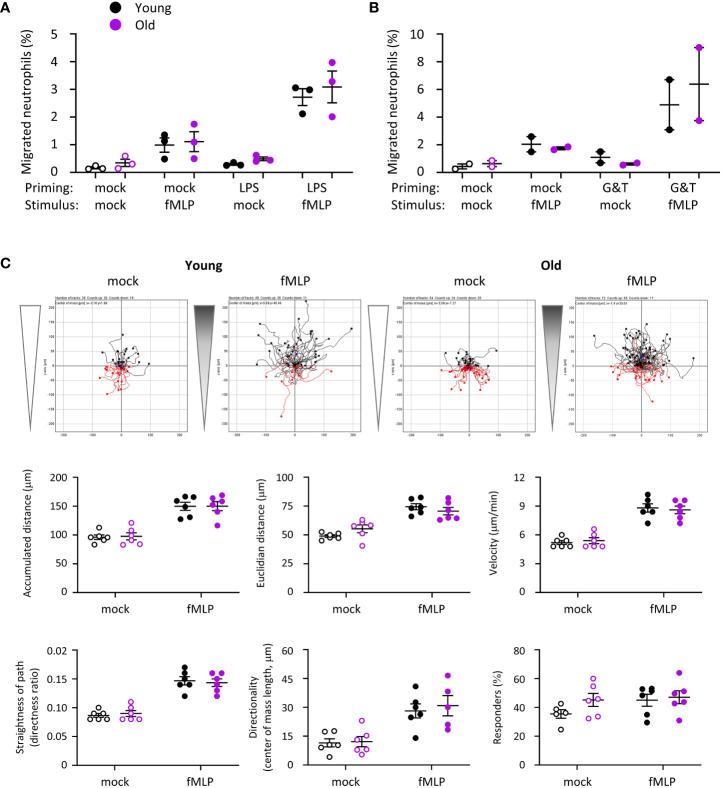
Hornigold K et al. Migration is normal in neutrophils from old mice. **(A, B)** Transwell assay. Bone marrow cells from young (8-10 weeks, black symbols) and old (24 months, purple symbols) mice were **(A)** primed with 1 μg/ml LPS or mock-primed for 90 min at 37°C, or **(B)** primed with 50 ng/ml GM-CSF and 20 ng/ml TNFα or mock-primed for 45 min, before placing them into the top well of transwell filters with 3 μM pores and stimulation with 1 μM fMLP, or mock-stimulation with buffer only, in the bottom well for 40 min at 37°C. Transmigrated and total neutrophils were quantitated by flow cytometry. Data are mean ± SEM of 3 independent experiments in **(A)** and 2 in **(B)**; each dot represents the mean of one experiment. **(C)** Ibidi chamber assays. Isolated neutrophils from young or old mice as in **(A)** were primed with 50 ng/ml GM-CSF and 20 ng/ml TNFα for 45 min and seeded into ibidi chambers coated with 20 μg/ml pRGD. Cells were allowed to adhere for 20 min at 37°C before establishing a gradient with 10 µM fMLP at one end of the chamber (filled symbols), or a mock-gradient with buffer only (open symbols), and neutrophils were live-imaged for 20 min (frames every 10 s) using an Olympus CellR microscope. Cells were tracked and migration quantitated using the ‘chemotaxis and migration’ plugin of ImageJ. Top panels show representative tracks from one experiment, other panels quantify the indicated parameters of cell speed and directionality obtained from ImageJ. Data are mean ± SEM of 6 independent experiments with tracks from 25-72 cells per condition in each experiment; each dot represents the mean of one experiment. Statistics in **(A)** and **(C)** were two-way ANOVA and revealed no differences between the ages.

Our *in vivo* experiments showing altered neutrophil recruitment in old mice suggested that neutrophil migration may be affected. In addition, reduced chemotaxis of neutrophils from old mice was previously reported (15, 28). Hence, we investigated neutrophil migration. Using transwell assays, we found no difference in spontaneous neutrophil migration or in chemotaxis to fMLP between young and old, regardless of whether the cells had been mock-primed, or primed with LPS or GM-CSF/TNFα (**Figures 6A, B**). To investigate the possibility of subtle defects in velocity or directionality, we used ibidi chamber chemotaxis assays, with an fMLP gradient ranging from 0 to 10 μM fMLP. Pilot experiments showed that these conditions elicited a clear chemotactic response without being saturating (**Supplemental Figure 2**). Nevertheless, neutrophils from old and young migrated equally well by all parameters tested (accumulated and Euclidian distance, velocity, straightness of path, directionality and % of responding cells), both during random migration and chemotaxis to fMLP (**Figure 6C**). This was seem both when the neutrophils were plated on the fibrinonectin-like surface poly-RGD (**Figure 6**) or on fibrinogen, although there was a trend towards increased chemotaxis of mock-primed cells from old mice on fibrinogen (**Supplemental Figure 3**). Therefore, in contrast to our expectations, migration is normal in neutrophils from old mice. It appears that the increased neutrophil recruitment *in vivo* stems from LPS-dependent changes in the tissue environment rather than neutrophil-intrinsic migratory properties.

Neutrophils from old mice had constitutively reduced L-selectin and increased FcγRIII cell surface levels, whereas the cell surface level of Mac1 was low in both ages. Priming with GM-CSF/TNFα led to upregulation of Mac1 and shedding of L-selectin in both ages equally and overcame the altered FcγRIII level (**Supplemental Figure 4**). In contrast, LPS priming increased the surface level of Mac1 and reduced L-selectin in neutrophils from old mice but not young, whereas it induced shedding of FcγRIII equally between the ages (**Supplemental Figure 4**). These data, together with the finding that LPS-treatment was able to prime fMLP-stimulated chemotaxis effectively (**Figure 6A**, p=0.0004), suggest that not the entire LPS signaling pathway, but only certain aspects of it are defective in neutrophils from old mice.

### Age-related dysregulation of the neutrophil proteome

**Figure 7:**
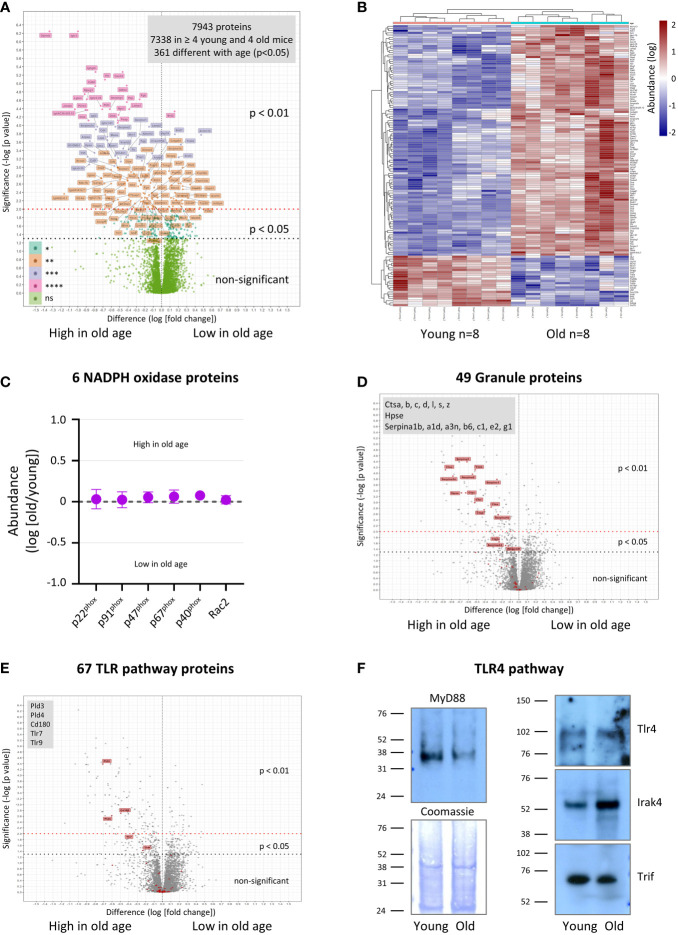
Hornigold K et al. Age-related dysregulation of the neutrophil proteome. Neutrophils were isolated to 95-98% purity from the bone-marrow of 8 young (8 weeks) and 8 old (24 months) mice, in 4 independent experiments with 2 young and 2 old mice per experiment, treated with the protease inhibitor DFP (7 mM) for 10 min at RT, washed twice, and frozen. Sample processing included protein digest and labeling with tandem mass-tags. Mass-tagged samples were combined, fractionated into 60 fractions, and analyzed by LC-MS mass spectrometry. Proteins were identified using Mascott software and their relative abundances determined using Proteome Discoverer software. **(A)** 7338 murine proteins were detected in at least 2 of 4 experiments (4 out of 8 mice per age) and analyzed using R software for differences in relative abundance between young and old. **(B)** Heatmap of proteins whose expression changed with p<0.01 between old and young mice. **(C-E)** Proteins were assigned to different classes and pathways by PANTHER pathway analysis and manual curation (see also **Supplemental Figures 5**–**8** and **Supplemental Table 1**). **(C)** Proteins of the NADPH oxidase complex. **(D)** Granule-lumen proteins. **(E)** TLR pathway proteins. The 49 granule-lumen proteins **(D)** and 67 TLR pathway proteins **(E)** identified (red) are plotted in the context of all other proteins (grey). Deregulated granule lumen proteins **(D)** and TLR pathway proteins **(E)** are shown by red flags and listed in the grey boxes. **(A-E)** Abundances are expressed as log(old/young). Statistical significance was assessed by two-sided t-test of log(young) vs log(old) with Benjamini-Hochberg false discovery rate correction for multiple comparisons of all 7338 quantified proteins. **(F)** Neutrophils were isolated and DFP-treated as in **(A)**, lysed, and total lysates pooled (4 mice per pool per age) and western blotted for the indicated proteins of the LPS/TLR4 signaling pathway. Coomassie staining was used as loading control. Blots shown are representative from one of two pools per age-group.

In order to begin to understand the origins of the functional impairments we observed, we compared the total proteomes of neutrophils from young and old mice using tandem mass-tag mass spectrometry. Overall, we identified murine 7943 proteins, and for 7338 of these we obtained enough data for a meaningful comparison between young and old (**Supplemental Table 1**). A surprisingly large number of proteins was not affected by age; only 361 proteins (5%) were expressed significantly differently, 207 higher in old age and 154 lower (**Figure 7A**, **Supplemental Figure 5** and **Supplemental Table 1**). A heatmap of the 139 most deregulated proteins (p<0.01) shows that the age-related changes were consistent between the 8 mice tested per age-group (**Figure 7B**). Among the dysregulated proteins, chromatin and RNA regulators were noticeably downregulated, whereas proteases, protease inhibitors and cell surface receptors were enriched in in old age (**Supplemental Figure 5**). Also upregulated were immunoglobulins, but this is almost certainly an artefact caused by the homing of immunoglobulin-producing plasma cells into the bone marrow in old age (45). As expected from our ROS assays, the components of the NADPH oxidase were all expressed to normal amounts, which reinforces that LPS priming but not overall NADPH oxidase capacity is affected (**Figure 7C**). In contrast, many of the anti-pathogen proteins stored in granules (46–48) were among the most upregulated proteins in old age, particularly proteases from the cathepsin family and protease inhibitors from the serpin family (**Figure 7D** and **Supplemental Figure 6**). Hence the defect in bacterial clearance observed *in vivo* was not caused by a simple decline in granule protein expression; it seems more likely that granule proteins are upregulated in old age to compensate for the impaired degranulation. Of note, not all granule proteins were deregulated. For example, the marker proteins for the main granule populations, myeloperoxidase, lactoferrin and gelatinase, were all expressed at normal levels (**Supplemental Figure 6**), suggesting that bulk granulopoiesis is normal in old age, but some granule proteins are upregulated specifically.

We looked into the expression of signaling and cytoskeletal proteins in more detail. Among signaling proteins upregulated in old age were membrane receptors (9%, 11 out of 117 identified), phospholipid-modifying proteins (7%, 5/71) and proteins from the TLR signaling pathways (7%, 5/67) (**Figure 7E**, **Supplemental Figures 7**, **8**). The deregulated membrane receptors comprised mainly Fc-receptors and integrins, but also the endosomal TLRs TLR7 and TLR9 (**Supplemental Figure 8A**). The phospholipid-modifying proteins comprised phosphoinositide kinases PI4K2a and phospholipases PLBD2, PLA2G15, PLD3 and PLD4 (**Supplemental Figure 7A**). PLD3 and PLD4 are of particular interest, as they were recently shown to not possess phospholipase activity, but rather to act as 5’ exonucleases which metabolize pathogenic nucleic acids in endosomes, the substrates of TLR7 and TLR9 (49). Apart from TLR7, TLR9, PLD3 and PLD4, only one other protein relevant to TLR pathways, the CD180 antigen, was upregulated in old age by mass spectrometry (3-fold, p=0.0019) (**Figure 7E** and **Supplemental Figure 7B**). CD180 is a TLR-like transmembrane protein that binds to TLRs, inhibiting signaling through the TLR4, TLR7 and TLR9 pathways (50, 51). Remarkably few proteins from the GTPase pathways (3%, 9/339), protein kinases (3%, 6/224) and protein phosphatases (0%, 0/86), ubiquitin pathways (2%, 4/262) and cytoskeletal proteins (3%, 6/189) were deregulated (**Supplemental Figures 8B**–**F** and **Supplemental Table 1**).

Among the deregulated pathways, TLR4 signaling is particularly relevant here, seen that LPS priming of the ROS response, phagocytosis and degranulation was impaired in neutrophils from old mice. We failed to obtain enough data for TLR4, the LPS receptor, for quantification by mass spectrometry. However, western blotting showed that TLR4 expression was unaltered by age (**Figure 7F**), in agreement with earlier reports on human neutrophils (8, 10). In contrast, expression of the adaptor protein MyD88, a key mediator of the TLR4 pathway, was consistently lower in neutrophils from old than young mice by western blot (5-fold, p<0.0001) (**Figure 7F**). Irak4, an important kinase in the TLR4 pathway, was upregulated in old age (4-fold, p=0.0028), whereas the adaptor protein Trif was unchanged (**Figure 7F**). The data on MyD88 and Irak4 expression suggest that western blotting may be a more sensitive readout for age-dependent changes than the tandem mass-tag mass spectrometry, which required many more sample preparation steps. In any event, both the upregulation of the inhibitory CD180 and downregulation of MyD88 may be sufficient to explain the decline in LPS priming capacity in old age.

### LPS/TLR4 pathway activity is impaired in neutrophils from old mice

**Figure 8:**
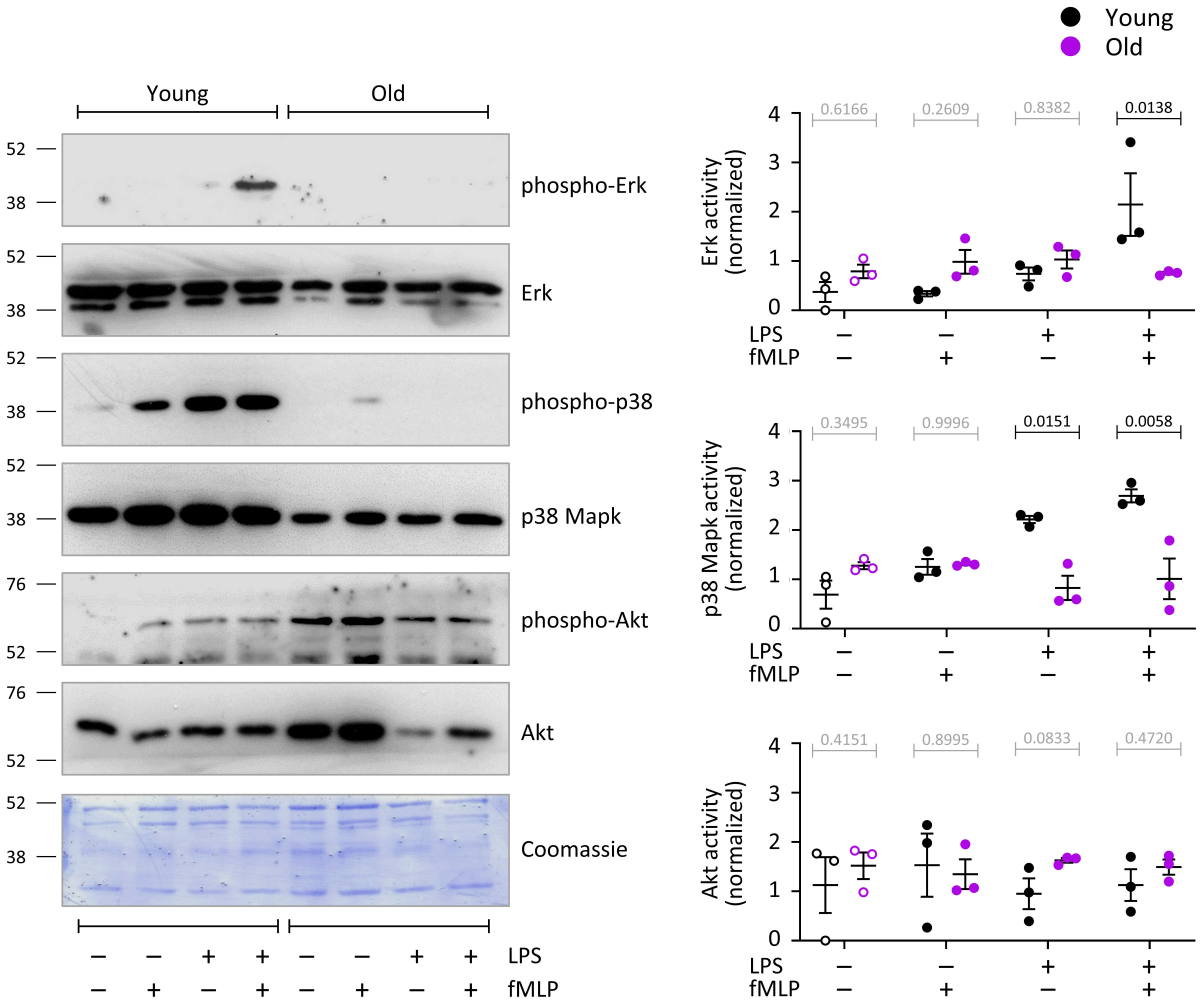
Hornigold K et al. LPS/TLR4 pathway activity is impaired in neutrophils from old mice. Neutrophils from young (8-10 weeks, black symbols) and old (24 months, purple symbols) mice were primed with 1 μg/ml LPS, or mock-primed with DPBS^++++^, and stimulated with 1 μM fMLP for 1 min. Total cell lysates were western blotted using antibodies for phosphorylated p38 Mapk, p42/44 Erk and Akt. Blots were stripped and reprobed for total p38 Mapk, p42/44 Erk and Akt. Blots were quantitated by ImageJ densitometry, and phospho-protein signals normalized to total-protein for each sample. Representative blots, and a coomassie-stained membrane as total loading control, are shown. Data are mean ± SEM of 3 independent experiments; each dot represents the mean of one experiment. Statistics are two-way ANOVA with Sidak’s multiple comparisons tests; black p-values are significant, grey p-values non-significant.

To investigate the LPS/TLR4 pathway further, we tested the effects of LPS priming and fMLP stimulation on the activities of Erk, p38 Mapk and Akt in neutrophils from young and old mice by phospho-western blot. LPS priming alone significantly (p=0.0095) activated p38 Mapk in neutrophils from young mice but not old (**Figure 8**). In mock-primed cells, fMLP stimulation (1 μM, 1 min) did not significantly activate any of the pathways. In contrast, in LPS-primed cells, fMLP stimulation led to robust further activation of p38 Mapk (p=0.0197) and induced the activation of Erk (p=0.0055), but only in neutrophils from young mice, not old (**Figure 8**). Akt was not significantly activated under the conditions tested here. Although there was a tendency for total protein levels of Erk and p38 Mapk to be higher in neutrophils from young mice than old, this did not reach significance throughout experiments. Together, these data show that LPS/TLR4 signaling through Erk and p38 Mapk is profoundly impaired in neutrophils from old mice.

### PIP_3_ production and PIP_2_ levels are reduced in neutrophils from old mice

**Figure 9:**
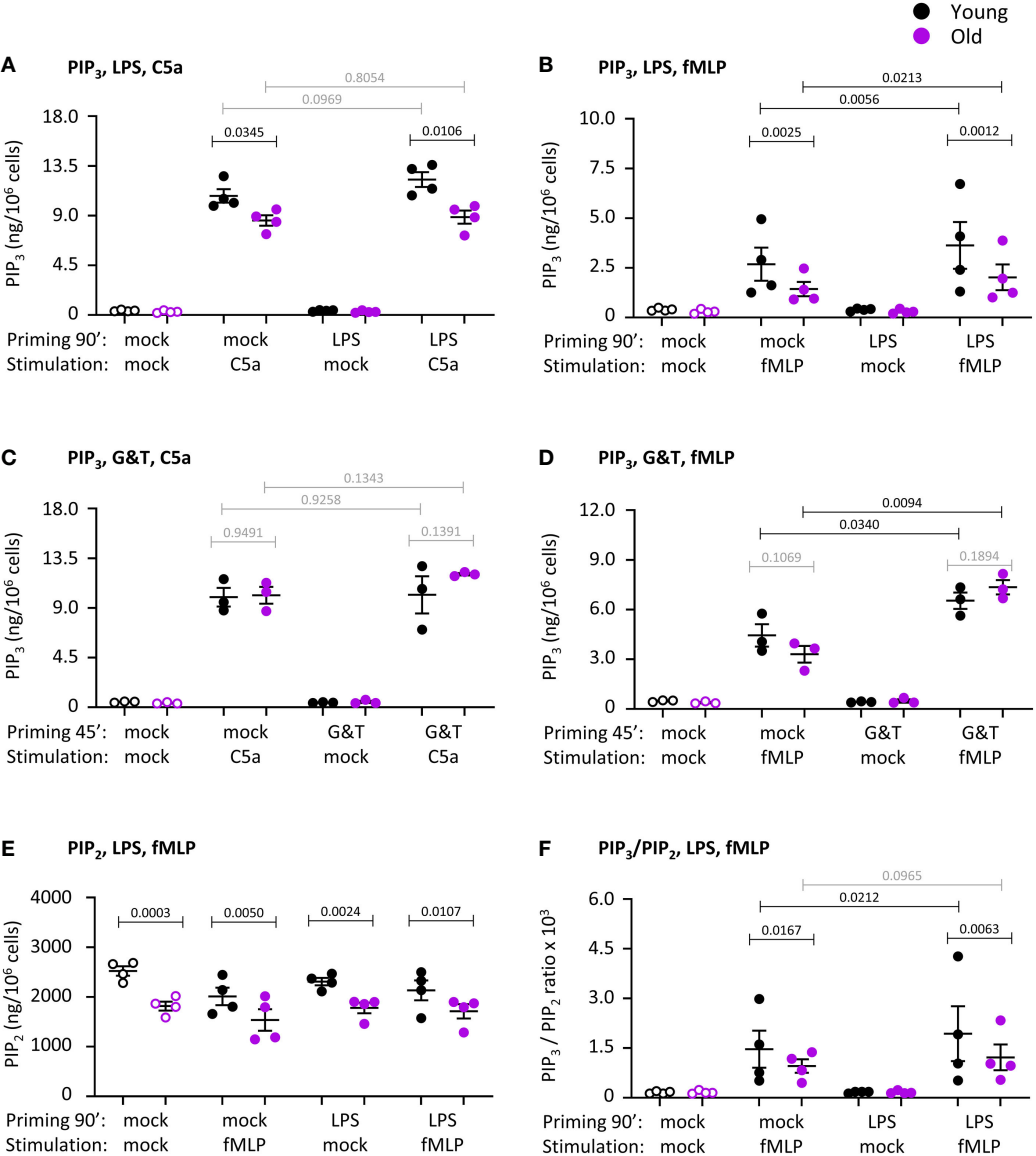
Hornigold K et al. PIP_3_ production and PIP_2_ levels are reduced in neutrophils from old mice. Neutrophils from young (8-10 weeks, black symbols) and old (24 months, purple symbols) mice were primed with 1 μg/ml LPS or mock-primed for 90 min **(A, B, E, F)**, or were primed with 50 ng/ml GM-CSF and 20 ng/ml murine TNFα or mock-primed for 45 min **(C, D)**, prior to stimulation with 25 nM C5a **(A, C)** or 3 µM fMLP **(B,D-F)**, or mock-stimulation, for 10 s. PIP_3_ **(A-D)** and PIP_2_ **(E)** were extracted and quantitated by lipid mass spectrometry using internal synthetic standards. Data in **(A, B, E, F)** are mean ± SEM of 4 independent experiments and data in **(C, D)** from 3; each dot represents the mean of one experiment. **(F)** Ratio of PIP_3_ over PIP_2_ from **(B)** and **(E)**. Statistics are two-way ANOVA with Sidak’s multiple comparisons test, for chemoattractant-stimulated cells only in **(A–D, F)**; black p-values are significant, grey p-values non-significant.

Production of the lipid second messenger PIP_3_ by class 1 PI3Ks is required for chemoattractant-stimulated ROS production (52). Hence we measured PIP_3_ in response to neutrophil stimulation with C5a or fMLP, either upon mock-priming or priming with LPS or GM-CSF/TNFα, using direct PIP_3_ measurement by lipid mass spectrometry (40). No PIP_3_ was detectable in mock-stimulated cells, even when cells were primed (**Figures 9A–D**). This was as expected (40), and confirms results from the ROS and degranulation assays showing that neutrophils from old mice are not pre-activated by inflamm-aging. Stimulation with C5a or fMLP induced rapid and robust PIP_3_ production, which was lower in old than young after cells had been mock-primed for 90 min (**Figures 9A–D**). Priming with LPS or GM-CSF/TNFα increased fMLP-stimulated PIP_3_ production, but had no effect on C5a-stimulated PIP_3_, and the fMLP-stimulated response remained lower in LPS-primed cells from old mice (**Figures 9A–D**).

PIP_3_ is produced by PI3K-mediated phosphorylation of the membrane phosphoinositide PIP_2_. Hence, we analyzed the same lipid mass spectrometry samples further to see whether the impaired PIP_3_ production might be caused by altered PIP_2_ levels. Indeed, PIP_2_ was constitutively reduced in neutrophils from old mice, and remained lower under all conditions tested ( **Figure 9E**). Yet even after taking into account the ratio of PIP_3_ to PIP_2_, as is commonly done in PIP_3_ mass-spectrometry (40, 41), neutrophils still showed the age-related reduction in fMLP-stimulated PIP_3_, suggesting that it was not solely a consequence of lower substrate availability. Taking into account PIP_2_, LPS-priming increased PIP_3_ production only in the young, revealing an age-related LPS-priming defect independent of PIP_2_ availability ( **Figure 9F**). Hence, neutrophils from old mice show reduced fMLP-stimulated production of PIP_3_ which is partially LPS-dependent, as well as having constitutively reduced levels of PIP_2_. The reduced LPS-primed PIP_3_ production might explain the impaired ROS response.

### Rac activity is normal in neutrophils from old mice

**Figure 10:**
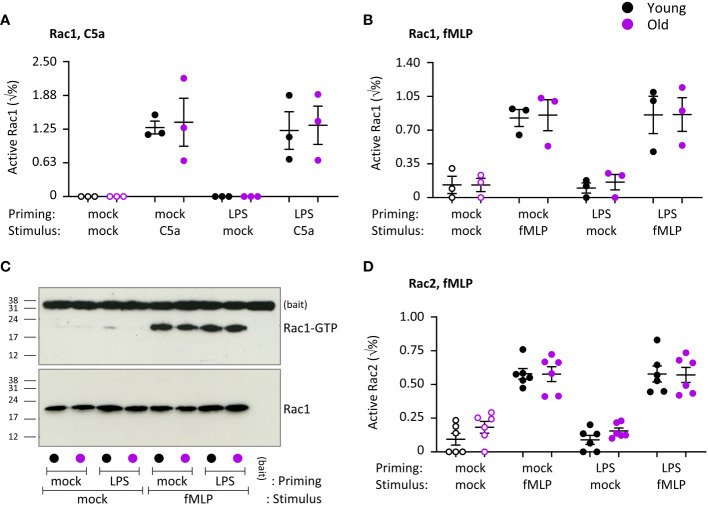
Hornigold K et al. Chemoattractant-stimulated Rac activity is normal in neutrophils from old mice. Neutrophils from young (8-10 weeks, black symbols) and old (24 months, purple symbols) mice were primed with 1 μg/ml LPS or mock-primed for 90 min, prior to stimulation for 10 s with 25 nM C5a **(A)** or 3 µM fMLP **(B-D)**, or mock-stimulation as indicated. Rac activity was assessed by Pak-CRIB pull down. GTP-Rac (active) and total Rac levels were analyzed by western blotting with Rac1 and Rac2 antibodies and quantified by densitometry using ImageJ. **(C)** Representative western blots from one experiment as in **(B)**. Data in **(A)** and **(B)** are mean ± SEM of 3 independent experiments and data in **(D)** of 6; each dot represents the mean of one experiment. Statistics were two-way ANOVA with Sidak’s multiple comparisons test and showed no differences between ages.

ROS production and migration require activation of the small GTPase Rac (53). Mouse neutrophils express two isoforms of Rac, Rac1 which confers directionality to migration, and Rac2 which confers the ability to migrate *per se* and is also required for ROS productions as an integral part of the NADPH oxidase complex. Seen that ROS production was impaired, we tested the activities of Rac1 and Rac2 in response to stimulation with C5a or fMLP, under mock-primed and LPS-primed conditions. Both Rac1 and Rac2 were robustly activated upon chemoattractant stimulation (p=0.0025 in A, p=0.0008 in B, p<0.0001 in D), but there was no difference between the ages. LPS priming had no effect on the activity of either Rac1 or Rac2 (**Figure 10**). Hence, as suggested by the normal migration properties of the neutrophils, the activities of Rac1 and Rac2 are not affected by age. These data imply furthermore that Rac activity is not related to the age-related decline in LPS-primed ROS production.

## Discussion

Our study shows that old mice have lower resistance to bacterial infection, their neutrophils have a reduced ability to kill bacteria, and all major neutrophil effector responses, with the notable exception of chemotaxis, are impaired, particularly in the context of LPS priming.

We showed that old mice have a lower capacity to clear pathogenic *E. coli* strain O18:K1 during an acute model of septic peritonitis. This infection model recapitulates the situation in humans, where the incidence of peritonitis increases with age to become a leading cause of mortality from sepsis for the elderly in intensive care units (4). Our data complement earlier reports on decreased immunity of old mice to cutaneous *S. aureus* infection (15), oral *S. typhimurium* infection and systemic *C. albicans* infection (17), showing that old mice have consistently reduced immunity to bacteria and fungi regardless of the infected organ. It would be valuable to perform a kinetic study in future to determine at which ages the immunity declines most.

In contrast to this consistent loss of innate immunity, the effects of age on neutrophil recruitment are much more varied, either reduced (15, 21, 22), or normal (54), or increased (19, 20) (23), depending on the affected organ and inflammatory or infectious agent. Congruently, we found that neutrophil recruitment was normal during aseptic peritonitis but elevated during LPS-induced peritonitis. The increased LPS-dependent recruitment is unlikely to be linked to the neutrophil-intrinsic defect in LPS priming, and more likely to systemically increased inflammatory chemokines/cytokines. A previous study used LPS to induce pulmonary neutrophil recruitment, which was also raised and accompanied by increased production of inflammatory cytokines (23), suggesting LPS may consistently increase recruitment in old mice regardless of the affected organ. A beautiful recent study used intravital microscopy to show that aberrant neutrophil trafficking in old mice is indeed caused by increased inflammatory cytokines in aged tissues rather than neutrophil-intrinsic defects (22). IL1β-stimulated neutrophil recruitment in the cremaster muscle of old mice was reduced due to increased reverse-transendothelial migration caused by mast cells in the aged tissue producing KC, leading to downregulation of the KC receptor CXCR2 on the neutrophil surface through agonist-induced internalization (22).

The finding by several studies that innate immunity declines in old mice regardless of effects on neutrophil recruitment (15, 17–20) suggested that neutrophil effector responses may be impaired. To study these effector responses, we required basal neutrophils that allowed us to induce specific pathways for priming and activation, which precluded the use of tissue-infiltrated neutrophils. We isolated mature neutrophils from the bone marrow rather than from peripheral blood in order to minimize any age-dependent differences in the proportions of freshly released and senescent neutrophils. Indeed, the peripheral pool of neutrophils is more senescent in old mice than young (increased CXCR4 and lower L-selectin/CD62L on the cell surface), due to reduced efferocytosis of aged neutrophils by macrophages in old mice (55). As mentioned before, neutrophil aging occurs in the circulation through priming by factors such as LPS and peptidoglycans produced by the gut microbiota (32). Our bone marrow-derived neutrophils showed no evidence of being pre-primed when we assayed ROS production, degranulation or migration. The neutrophils from old mice did, however, show a reduction in L-selectin and increase in FcγRIII levels on their surface in the basal state (on ice), raising the possibility that they are partially primed. Alternative explanations that remain to be tested could be altered total levels or altered shedding of these proteins. The cell surface level of Mac1 was as low in basal neutrophils of both ages, as was the secretion of gelatinase granules (see below), arguing against a primed state in old age. Overall, it appears our approach effectively prevented confounding the effects of senescence and organismal age, but a more in-depth analysis of priming state may be required in the future.

ROS production by neutrophils from elderly humans is impaired in response to a range of priming agents and stimuli (7–10), whereas ROS production by neutrophils from old mice is more varied, either normal or reduced depending on stimulus (24, 25). PMA-stimulated ROS production is normal in neutrophils from both elderly humans (10) and old mice (our data), and all subunits of the NADPH oxidase are expressed at normal levels in old age, so aging clearly does not affect the NADPH oxidase itself but rather the upstream signaling pathways. The ROS response was normal upon priming with GM-CSF/TNFα, but impaired after LPS priming both upon stimulation with C5a and fMLP. Therefore, bone-marrow derived mouse neutrophils are a good model for the age-related decline in ROS production, particularly when associated with LPS priming. Human neutrophils are usually isolated from the periphery rather than the bone marrow, and like in mice, the peripheral population is more senescent (CD62L^lo^) in old people than young from priming in the vasculature (56). The reduction in the GM-CSF primed ROS response that is commonly seen in elderly humans may therefore be a consequence of senescence rather than a neutrophil-intrinsic defect.

Phagocytosis is generally reduced in neutrophils from elderly humans (12, 13), whereas it was reported to be more variable in neutrophils from old mice, either reduced (27) or normal (15). Our assays showed an overall reduction in the phagocytosis of both *E. coli* and zymosan yeast particles. The number of *E. coli* or zymosan particles taken up was impaired in neutrophils from old mice after LPS priming, and there was also a decline in the proportion of phagocytosing neutrophils which was independent of LPS. This partial LPS dependence, together with the need to assess several parameters of phagocytosis to document a phenotype, may explain why some previous studies failed to observe an age-dependent decline in phagocytosis.

Neutrophil priming occurs through degranulation. The fusion of granules with the plasma membrane upregulates receptors stored on granules to the plasma membrane, readying the cell for stimulation, as well as causing the release of proteases and other anti-pathogen factors from the granule lumen into the extracellular space. Surprisingly little information on the effects of age on degranulation exists, both in human and mouse. The blood plasma of elderly humans contains more cleavage products of neutrophil elastase, an azurophil granule lumen protease, and neutrophils from elderly people have higher cell surface levels of CD63, a membrane protein stored on azurophil granules (11), and show higher elastase activity after phagocytosis of *S. pneumoniae* (57). However, the former may be a consequence of neutrophil senescence or death and the latter may reflect phagocytic capacity rather than degranulation. Direct degranulation assays with isolated neutrophils from the elderly have to our knowledge never been done. We show that, gelatinase release was low in basal neutrophils, with no difference between the ages. As the secretion of gelatinase granules is particularly sensitive to priming (46, 47), this further supports the notion that bone marrow-derived neutrophils from old mice are not primed. Neutrophils from old mice showed reduced degranulation of azurophil granules (induced by *E. coli*) as well as gelatinase granules (induced by LPS/fMLP), and the latter defect had again both LPS-dependent and LPS-independent elements. The overall capacity to degranulate was normal when depolymerization was forced with cytochalasin B. It seems possible that neutrophils from old mice are more reticent to depolymerize their cortical actin ring, making granule fusion harder. The age-related decrease in degranulation contrasted with the marked upregulation of granule lumen proteins from the cathepsin and serpin families. Hence, the decline in bacterial clearance *in vivo* is not caused by reduced expression of granule proteins. Evidently, the upregulation of cathepsins is insufficient to protect old mice during bacterial infection, but possibly the upregulation of serpins, which inhibit proteases, might contribute to the age-related decline in immunity. It would be of interest to investigate why cathepsins and serpins are selectively deregulated among granule proteins. In addition to these granule proteins, several other proteases and protease inhibitors were upregulated in old age. It would also be interesting to study the subcellular localizations of these proteins in the future.

NET release by neutrophils from elderly humans is impaired in response to many stimuli (7–10), whereas like the other neutrophil responses, this is reportedly more variable in neutrophils from old mice, either reduced upon stimulation with TLR2 ligands (24), normal after stimulation with PMA (26), or increased upon mitochondrial oxidative stress (25). We found normal NET release in response to *E. coli* in old age, but reduced *S. aureus*-stimulated NETs. The lack of effect of age on *E. coli*-induced NET release suggests that the rapid killing of *E. coli* by young neutrophils, which was impaired in the old, was a consequence of responses such as phagocytosis and degranulation rather than NET release. LPS priming had hardly any effect, so this defect is independent of LPS. This was surprising, as ROS production is often strongly linked with NETs, and we saw an age-dependent decline in LPS-primed ROS production. However, NET release can also occur independently of ROS production, through mechanisms which remain incompletely understood (58). Our proteomic found no dysregulated expression of NETs-related proteins such as histones, PADI4 and elastase, in contrast to their previously reported up- or downregulated transcription (26), suggesting either that transcriptomics is a more sensitive readout or that protein production or stability overrides mRNA levels in these cases. Future work will be required to elucidate which NET pathways are impaired.

We showed that both random migration and fMLP-stimulated chemotaxis were normal in neutrophils from old mice, regardless of whether the cells were mock-primed or primed with LPS or GM-CSF/TNFα, both in transwell assays and in imaging-based ibidi assays on pRGD and fibrinogen surfaces. Therefore, the increased peritoneal neutrophil recruitment in response to LPS *in vivo* was clearly not a consequence of an altered intrinsic ability of neutrophils to migrate. Rather, our results support the recent study discussed above, which showed that altered neutrophil recruitment in old age is caused by extrinsic, tissue-derived factors (22). Other mouse studies reported normal or elevated spontaneous neutrophil migration, and reduced chemotaxis in old age (19, 28). Chemotaxis of neutrophils from elderly humans is also reduced, despite normal surface levels of major chemoattractant receptors (8, 11). We can only assume that the difference between our study and other mouse neutrophil studies is due to different assay conditions. However, we employed similar transwell assays and additionally the more sophisticated ibidi chamber, which if anything should have made it more likely to detect defects in migration. Using the ibidi assay under the same conditions, we could confidently detect differences in migration speed of 20% in other projects (unpublished observation), so assay sensitivity was not limiting. It is possible that the age-related impairment is specific to certain chemoattractants, as we employed fMLP, whereas as the previous studies used KC, despite the surface level of the KC receptor CXCR2 being reduced in neutrophils from old mice (19). We found altered cell surface levels of L-selectin and FcγRIII in neutrophils from old mice, and altered effects of LPS priming on Mac1 and L-selectin surface levels. It is therefore possible that more detailed future analysis of LPS-primed migration, particularly under shear-stress conditions, might reveal age-related defects.

Neutrophil proteomics had been done in multiple previous studies, mostly studies on subcellular fractions such as the various granule subsets, but also functional proteomics such as phospho-proteomics or changes associated with diurnal rhythms or neutrophil-dependent diseases such as chronic granulomatous disease or leukocyte adhesion deficiency (47, 48, 59, 60). One study compared the proteomes of neutrophils from newborn and adult humans, showing low abundance in newborns of proteins related to the proteasome, transendothelial migration and NETosis, as well as several granule proteins including elastase and myeloperoxidase (61). However, to our knowledge neutrophil proteomes have never been compared between young and old adults, neither in humans nor mice. A recent study compared transcriptomics, metabolomics and lipidomics of bone marrow-derived neutrophils from male and female old and young mice (26). Sex-differences outweighed age-differences, and age-dependent changes were seen only by transcriptomics but not metabolomics or lipidomics (26). Like our proteomic analysis, transcriptomics found chromatin regulators among the most downregulated genes in old age, associated with higher chromatin compaction. In addition, the mRNAs of several cell-cycle regulators were downregulated (26) which we did not see on the protein level, whereas we found reduced expression of many RNA regulators. Like our proteomics, the transcriptomics also detected the upregulation of several granule proteins in old age (26), but did not detect the increased levels of serpins. The transcriptomics also did not note any upregulation of membrane receptors, phospholipid-modifying or TLR pathway genes in old age. Overall however, the congruence between the two studies regarding chromatin regulators and granule proteases shows robust age-related changes.

Our proteomic analysis of the TLR pathways revealed an upregulation in old age of CD180, a transmembrane protein that inhibits signaling through TLR4 and other TLR pathways (50, 51). Furthermore, we found reduced expression of MyD88 by western blotting. MyD88 expression is also reduced in neutrophils from elderly humans (8). MyD88 confers one of the two major arms of the TLR4 signaling pathway, leading to the activation of NF-κB-responsive genes for the production of inflammatory cytokines (62). As CD180 and Myd88 are important regulators of the TLR4 pathway, their deregulation is likely to contribute to the impaired LPS priming in old age. It is important to note however that LPS-primed chemotaxis and shedding of FcγRIII were normal, meaning that not the entire LPS priming pathway is defective. Perhaps the MyD88-independent arm of the TLR4 pathway which is regulated by Trif (62) remains intact, as we found normal Trif levels. Moreover, aging will not only affect the expression but also the subcellular localization and activity of TLR4 pathway components. Evidence for this was seen in neutrophils from elderly humans, where the LPS-induced localization of TLR4 and Irak1 to lipid rafts is reduced (8). We demonstrate profoundly impaired activation of Erk and p38 Mapk in LPS-primed neutrophils from old mice. A more detailed future characterization of LPS/TLR4 signaling will be required to elucidate the mechanisms through which this pathway is impaired. Furthermore, our proteomics showed that components of other TLR signaling pathways, notably the endosomal TLR7 and TLR9, as well as exonucleases PLD3 and PLD4 which metabolize substrates of endosomal TLRs (49), are among the most upregulated proteins in old age. Therefore, in addition to the TLR4 pathway, the effects of age on signaling through these endosomal TLRs would merit investigation.

We investigated the activity of class I PI3K, an important effector of the LPS/TLR4 pathway (63). PI3K activity was previously reported to be constitutively increased in neutrophils from elderly humans, and inhibition of PI3Kγ or PI3Kδ restored speed and accuracy during neutrophil chemotaxis (11). However, in that study PI3K activity was determined through phosphorylation of the regulatory PI3K subunit (11), an indirect proxy that can be a poor indicator of activity. We used direct lipid mass spectrometry to measure PI3K activity (40), which revealed a partially LPS-dependent reduction of chemoattractant-stimulated PIP_3_ production. Hence, class I PI3K activity is reduced rather than increased in old age, both upon prolonged incubation of cells at 37°C and upon LPS priming, but not after GM-CSF/TNFα priming, which was unaffected. Furthermore, the neutrophil isoform PI3Kγ specifically determines the proportion of neutrophils that migrate by chemokinesis (64), whereas we only tested spontaneous migration and chemotaxis. In view of these PIP_3_ results, it would be interesting to test chemokinesis.

The age-dependent loss of signaling through the LPS/TLR4 pathway is not specific to neutrophils. Loss of TLR4 and other TLR signaling pathways with age has also been shown in monocytes, macrophages and dendritic cells. Peritoneal or spleen-derived macrophages from old mice showed reduced production of inflammatory cytokines in response to LPS despite normal levels of TLR4, with reduced activation of p38^Mapk^ and Jnk (65, 66). Similarly, LPS-induced maturation of dendritic cells is reduced in old mice (67, 68), and splenic dendritic cells produce less TNFα upon LPS stimulation despite normal TLR4 levels (67). Furthermore, peripheral blood monocytes showed reduced production of inflammatory cytokines in response to stimulation of TLR1/2, and lower TLR1 surface levels in old age (69), as well as impaired NF-κB activity in response to TLR5 ligands, despite higher TLR5 expression and TLR5-dependent activation of p38 Mapk and Erk (70). Hence, age-related impairments of TLR pathways are seen in the mouse throughout different types of innate immune cells. How the reduced TLR functions in each of these cell types contribute to the impaired innate immunity of old mice *in vivo*, however largely remains to be demonstrated.

Our study suggests that mice are an appropriate model for studying the age-related decline human neutrophil function, particularly regarding the impaired LPS/TLR4 pathway. Age-related impairments in ROS production, degranulation, phagocytosis and PIP_3_ production were all partially LPS-dependent. This LPS/TLR4 pathway dependence resolves some of the previous controversy regarding the effects of age on murine neutrophils. No other study specified the use of endotoxin-free reagents, and only three recent papers (22, 24, 26) described the health status of the mice, specifying pathogen-controlled conditions. This suggests that neutrophils may have been in varying states of priming, especially in some older studies. Finally, the impaired ability to kill bacteria *in vitro* and reductions in neutrophil ROS production, phagocytosis, degranulation and NET formation that we demonstrated here may all contribute to the decline in antibacterial immunity *in vivo*. Future studies will need to evaluate the contribution of each neutrophil response to this impaired innate immunity through the use of genetic deficiencies, neutrophil depletion and pharmacological inhibitors.

## Data availability statement

The proteomics data presented in this study are deposited in the PRIDE repository, accession number PXD035577.

## Ethics statement

The animal study was reviewed and approved by the Babraham Institute Animal Welfare Ethical Review Body and the British Home Office.

## Author contributions

KH, JYC, PAM, LC, SAC, CP and DO devised, conducted and analyzed experiments, KEA and AS-P devised and analyzed experiments, PTH devised experiments, HCEW obtained funding, devised and analyzed experiments and wrote the paper. All authors contributed to the article and approved the submitted version.

## Funding

PM is the recipient of a targeted PhD studentship from the BBSRC. SC is the recipient of a targeted PhD studentship from the MRC. CP was the recipient of a DTP PhD studentship from the BBSRC. This project was funded by Institute Strategic Programme Grant BB/P013384/1 from the BBSRC to the Signalling Programme at the Babraham Institute.

## Acknowledgments

We thank the staff of the Babraham Biological Support Unit and the Imaging and Flow Cytometry facilities for their dedicated and highly skilled support.

## Conflict of interest

The authors declare that the research was conducted in the absence of any commercial or financial relationships that could be construed as a potential conflict of interest.

## Publisher’s note

All claims expressed in this article are solely those of the authors and do not necessarily represent those of their affiliated organizations, or those of the publisher, the editors and the reviewers. Any product that may be evaluated in this article, or claim that may be made by its manufacturer, is not guaranteed or endorsed by the publisher.

## Abbreviations

AUC, area under the curve; fMLP, formyl-methionyl-leucyl-phenylalanine; HPLC-MS, high-performance liquid-chromatography mass spectrometry; KC, keratinocyte-derived chemokine; LPS, lipopolysaccharide; NETs, neutrophil extracellular traps; PI3K, phosphoinositide 3 kinase; PMA, phorbol 12-myristate 13-acetate; PIP_3_, phosphatidylinositol (3,4,5)-trisphosphate; PIP_2_, phosphatidylinositol bisphosphate; ROS, reactive oxygen species; TLR, toll-like receptor; TNFα, tumor necrosis factor alpha.
