# Supplementary material for: Age-related decline in the resistance of mice to bacterial infection and in LPS/TLR4 pathway-dependent neutrophil responses
Source: Front Immunol. 2022 Aug 24;13:888415. doi: 10.3389/fimmu.2022.888415 (PMC9450589; doi:10.3389/fimmu.2022.888415)
Supplement: Supplementary file 2 [file Presentation_1.pdf]

frontiers

Supplementary Material

Age-related decline in the resistance of mice to bacterial infection and in LPS/TLR4 pathway-dependent neutrophil responses

Kirsti Hornigold[1], Julia Y. Chu[1], Stephen Chetwynd[1], Polly A. Machin[1], Laraine Crossland[1], Chiara Pantarelli[1], Karen E. Anderson[1], Phillip T. Hawkins[1], Anne Segonds-Pichon[2], David Oxley[3], Heidi C. E. Welch[1,*]

[1] Signalling Programme, [2] Bioinformatics Facility, and [3] Proteomics Facility, The Babraham Institute, Cambridge, United Kingdom

Short title: Age-related decline in neutrophil responses

Supplemental Figure Legends

Supplemental Figures 1-8

Supplemental Table 1 (separate Excel file)

Supplemental Figure Legends

Supplemental Figure 1. Priming of ROS production with TNFα or GM-CSF or both. Isolated neutrophils from young (8-10 weeks) mice were primed with 5 ng/ml TNFα or 100 ng/ml GM-CSF or both, as indicated, for 45 min at 37°C, prior to stimulation with 3 μM fMLP (closed symbols) or mock-stimulation (open symbols). ROS production was measured by real-time chemiluminescence assay with luminol and HRP for extra- and intracellular ROS. Data are mean ± SEM of 5 independent experiments; each dot is the mean AUC of the ROS response from one experiment. Statistics are two-way ANOVA with Sidak's multiple comparisons tests on log-transformed raw data.

Supplemental Figure 2. fMLP dose response of neutrophil chemotaxis. Neutrophils from young (8-10 weeks) mice were primed with 50 ng/ml GM-CSF and 20 ng/ml TNFα for 45 min and seeded into ibidi chambers coated with 1 mg/ml fibrinogen. Cells were allowed to adhere for 20 min at 37°C before establishing a gradient with 0, 1, 10 or 100 μM fMLP as the highest concentration, as indicated. Neutrophils were live-imaged for 20 min (frames every 10 s) at the steepest part of the gradient using an Olympus CellR microscope. Cells were tracked using the 'chemotaxis and migration' plugin of ImageJ. **(A)** Representative tracks (top) and rose plots (bottom) from one experiment are shown. **(B)** Quantification of the indicated parameters of cell speed and directionality by ImageJ. Data are mean ± range of two independent experiments.

Supplemental Figure 3. Migration on fibrinogen is normal in neutrophils from old mice. Neutrophils from young (8-10 weeks, black symbols) and old (24 months, purple symbols) mice were primed with 50 ng/ml GM-CSF and 20 ng/ml TNFα for 45 min (filled symbols), or were mock-primed (open symbols), and seeded into ibidi chambers coated with 1 mg/ml fibrinogen. Cells were allowed to adhere for 20 min at 37°C before establishing a gradient with 10 μM fMLP as the highest concentration, and neutrophils were live-imaged for 20 min (frames every 10 s) at the steepest part of the gradient using an Olympus CellR microscope. Cells were tracked using the 'chemotaxis and migration' plugin of ImageJ. **(A)** Representative tracks (top) and rose plots (bottom) are shown. **(B)** Quantification of indicated parameters of cell speed and directionality by ImageJ. Data are mean ± SEM of 15-22 mock-primed cells and 52-75 primed cells per condition from one of two independent experiments. Statistics were two-way ANOVA with Sidak's multiple comparisons and showed no differences between ages, as indicated by p-values in grey.

Supplemental Figure 4. Deregulated receptor surface levels in neutrophils from old mice. Bone marrow cells from young (8-10 weeks, black symbols) and old (24 months, purple symbols) mice were were either kept on ice (basal), or were primed with 1 μg/ml LPS or with 20 ng/ml TNFα and 50 ng/ml GM-CSF (G&T) for 45 min at 37°C, as indicated. Cells were stained with antibodies for Ly6G, Mac1 (CD11b), FcγRIII (CD16), and L-selectin (CD62L), and analyzed by flow cytometry. Neutrophils were identified by Ly6G^{hi}, CD11b^{hi} staining, and the mean fluorescence intensity (mfi) of Mac1, L-selectin and FcγRIII levels on the neutrophil surface quantitated using FlowJo. **(A)** Gating strategy. **(B)** Quantification of receptor levels on the neutrophil surface. Data are mean ± SEM of 3-4 independent experiments; each dot represents the mean of one experiment. Statistics were two-way ANOVA with Sidak's multiple comparisons; black p-values are significant, grey p-values non-significant.

Supplemental Figure 5. Deregulated protein expression in neutrophils from old mice. The total proteome of neutrophils from 8 young (8 weeks) and 8 old (24 months) mice was identified by tandem mass-tag mass spectrometry and analyzed for abundance in young and old as described in Figure 7.

(A) Proteins which were expressed significantly less in neutrophils from old mice. **(B)** Proteins which were expressed significantly more in neutrophils from old mice. Abundances are expressed as log(old/young). Statistical significance was assessed by two-sided t-test of log(young) vs log(old) with Benjamini-Hochberg false discovery rate correction for multiple comparisons on all 7338 quantified proteins. Colors denote different classes of proteins, as indicated, as determined by PANTHER pathway analysis and manual curation.

Supplemental Figure 6. Deregulated expression of granule lumen proteins in neutrophils from old mice. The total proteome of neutrophils from 8 young (8 weeks) and 8 old (24 months) mice was analyzed was identified by tandem mass-tag mass spectrometry and analyzed for abundance in young and old as described in Figure 7. Plotted are the 49 granule lumen proteins identified by PANTHER pathway analysis and manual curation based on the granule literature. Abundances are expressed as log(old/young). Statistical significance was assessed by two-sided t-test of log(young) vs log(old) with Benjamini-Hochberg false discovery rate correction for multiple comparisons on all 7338 quantified proteins. Light purple symbols denote proteases and other types of anti-pathogen proteins, dark purple symbols show protease inhibitors, and grey diamonds show proteins which were detected but not in sufficient samples for quantification.

Supplemental Figure 7. Deregulated expression of phospholipid-modifying proteins and proteins from the TLR signaling pathways in neutrophils from old mice. The total proteome of neutrophils from 8 young (8 weeks) and 8 old (24 months) mice was analyzed was identified by tandem mass-tag mass spectrometry and analyzed for abundance in young and old as described in Figure 7. **(A)** Plotted are the 64 phospholipid-modifying proteins (phospholipid kinases, phosphatases and lipases) identified by PANTHER pathway analysis and manual curation. **(B)** Plotted are the 67 proteins from TLR signaling pathways identified by PANTHER pathway analysis and manual curation based on https://reactome.org/content/detail/R-HSA-168898 (Toll-like Receptor Cascades). Abundances are expressed as log(old/young). Statistical significance was assessed by two-sided t-test of log(young) vs log(old) with Benjamini-Hochberg false discovery rate correction for multiple comparisons on all 7338 quantified proteins. Grey diamonds show proteins which were detected but not in sufficient samples for quantification.

Supplemental Figure 8. Deregulated expression of selected protein classes and pathways in neutrophils from old mice. The total proteome of neutrophils from 8 young (8 weeks) and 8 old (24 months) mice was analyzed was identified by tandem mass-tag mass spectrometry and analyzed for abundance in young and old as described in Figure 7. Plotted are the **(A)** 117 membrane receptors, **(B)** 339 GTPases pathway proteins, **(C)** 224 protein kinases, **(D)** 87 protein phosphatases, **(E)** 242 ubiquitin pathway proteins and **(F)** 189 cytoskeletal proteins identified by PANTHER pathway analysis and manual curation (red dots and boxes), on the background of all 7338 quantified proteins (grey dots). Grey boxes list proteins that are significantly upregulated in old age (left-hand side of graphs), or downregulated in old age (right-hand side of graphs). Abundances are expressed as log(old/young). Statistical significance was assessed by two-sided t-test of log(young) vs log(old) with Benjamini-Hochberg false discovery rate correction for multiple comparisons on all 7338 quantified proteins.

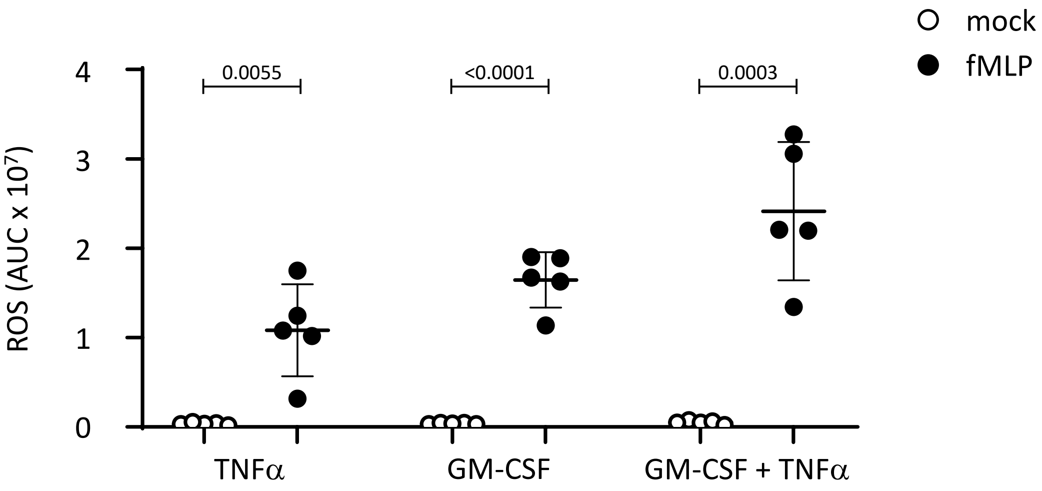

Supplemental Figure 1. Hornigold K *et al.* GM-CSF and TNFα-primed ROS production

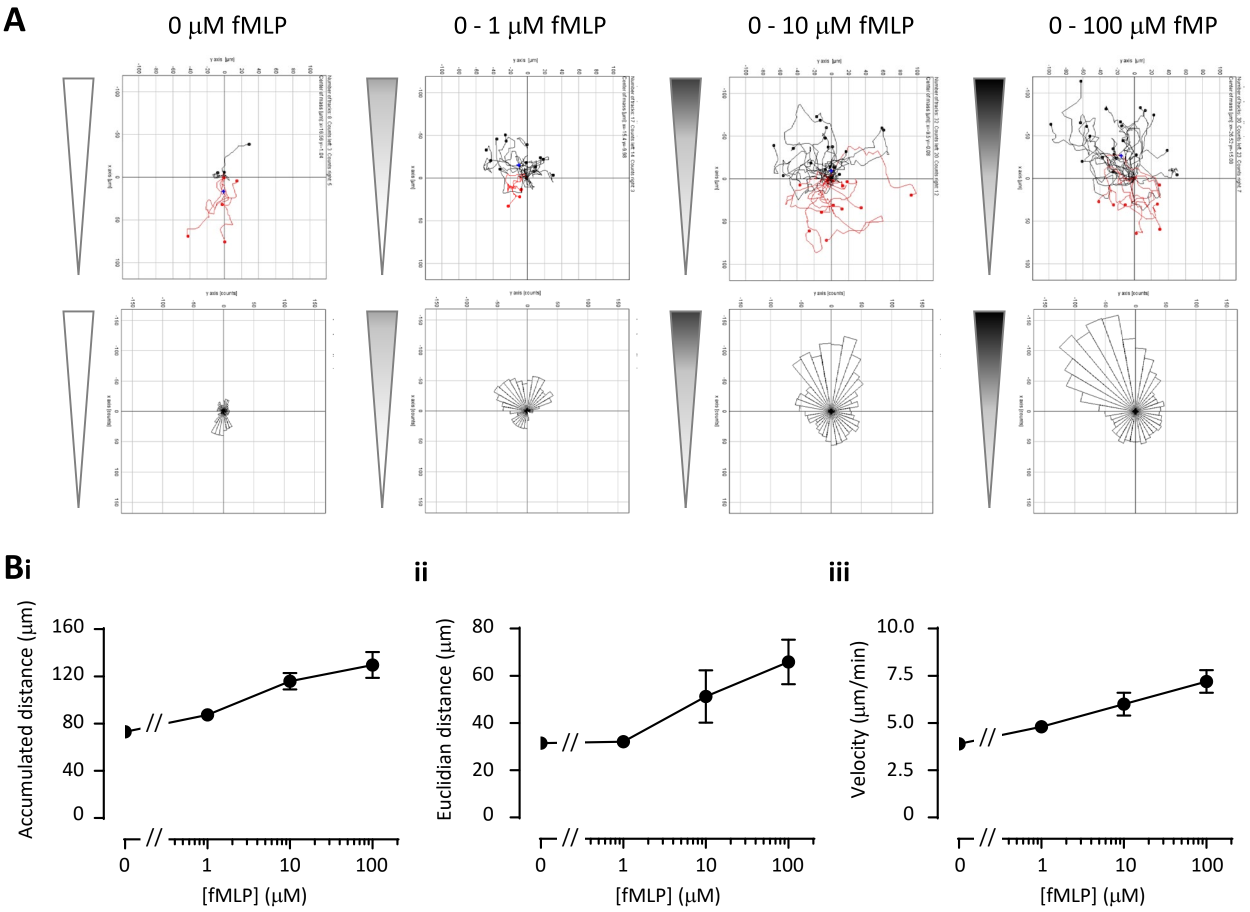

Supplemental Figure 2. Hornigold K *et al.* fMLP dose response of neutrophil chemotaxis

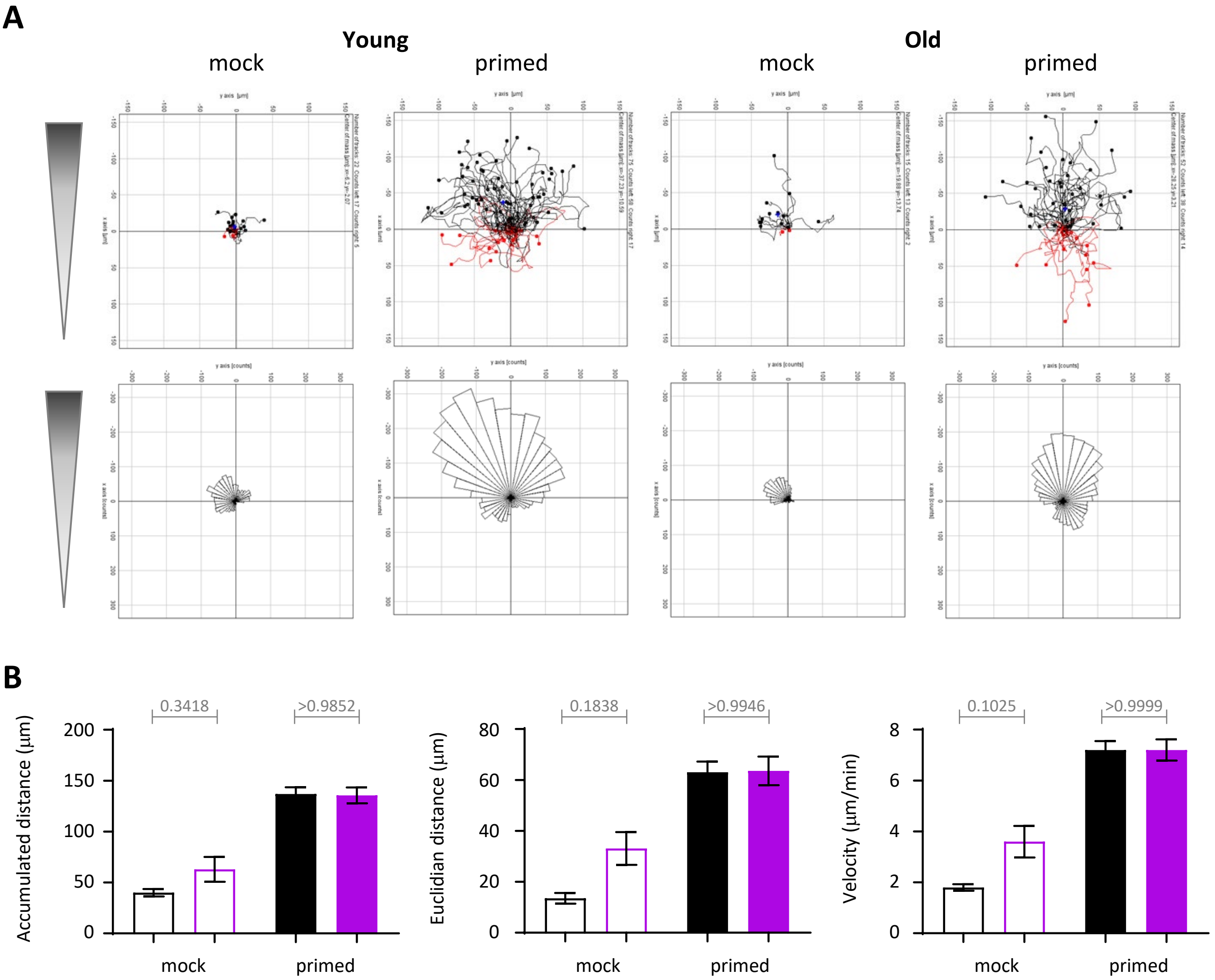

Supplemental Figure 3. Hornigold K *et al.* Normal neutrophil chemotaxis on fibrinogen in old age

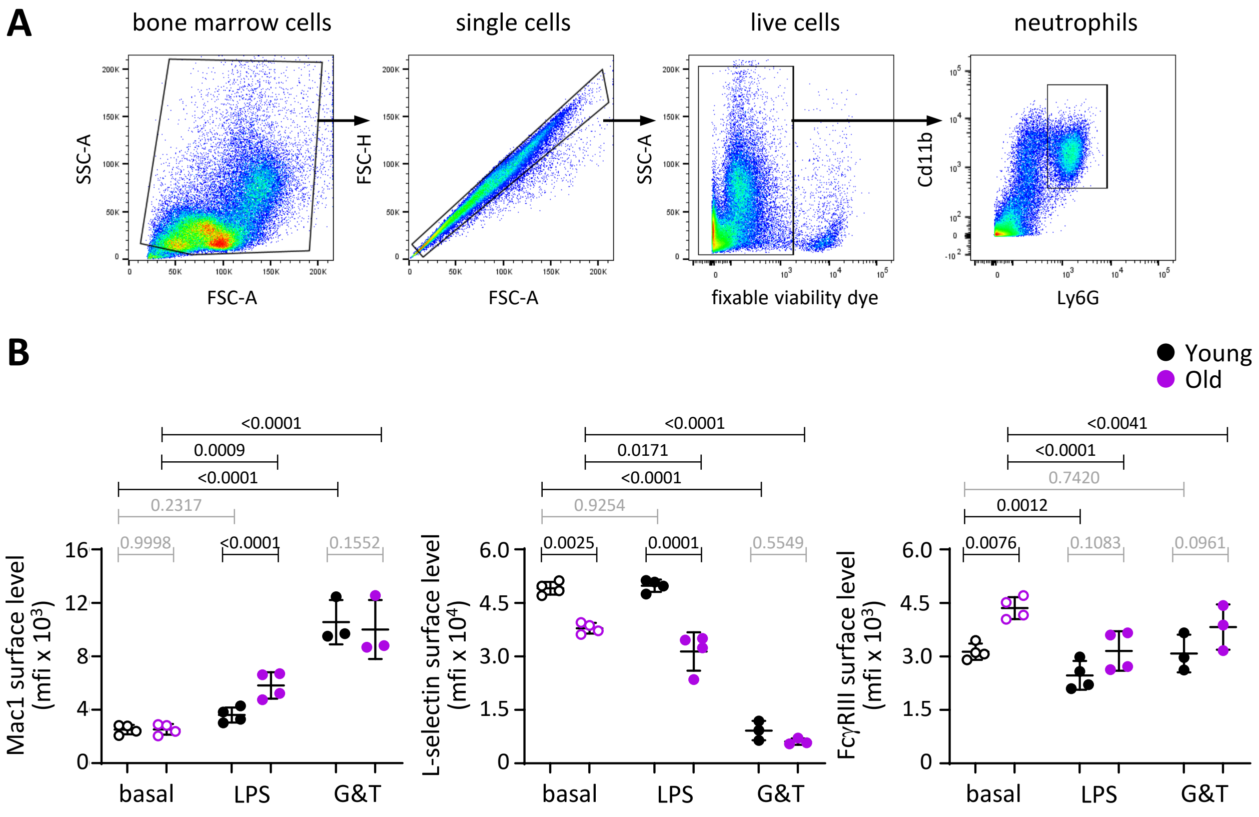

Supplemental Figure 4. Hornigold K *et al.* Cell surface levels of receptors

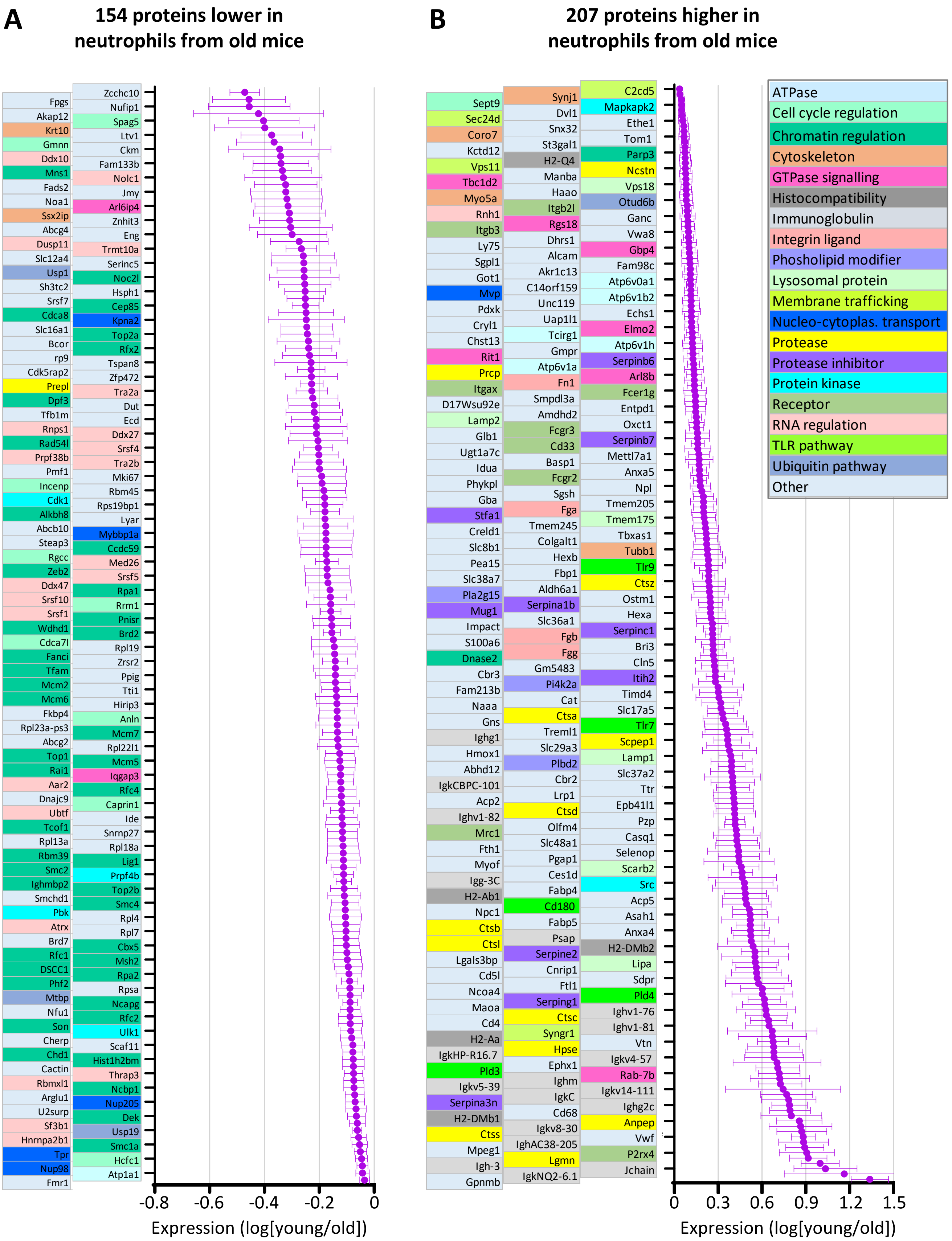

Supplemental Figure 5. Hornigold K *et al.* Altered protein expression with aging

49 granule lumen proteins

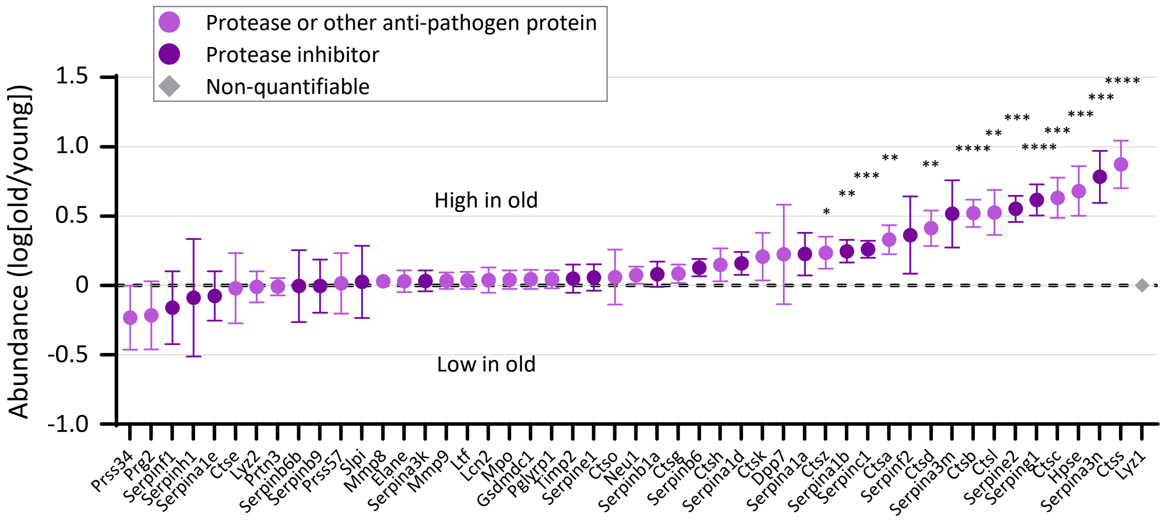

Supplemental Figure 6. Hornigold K *et al.* Deregulated expression of granule proteins in old age

A

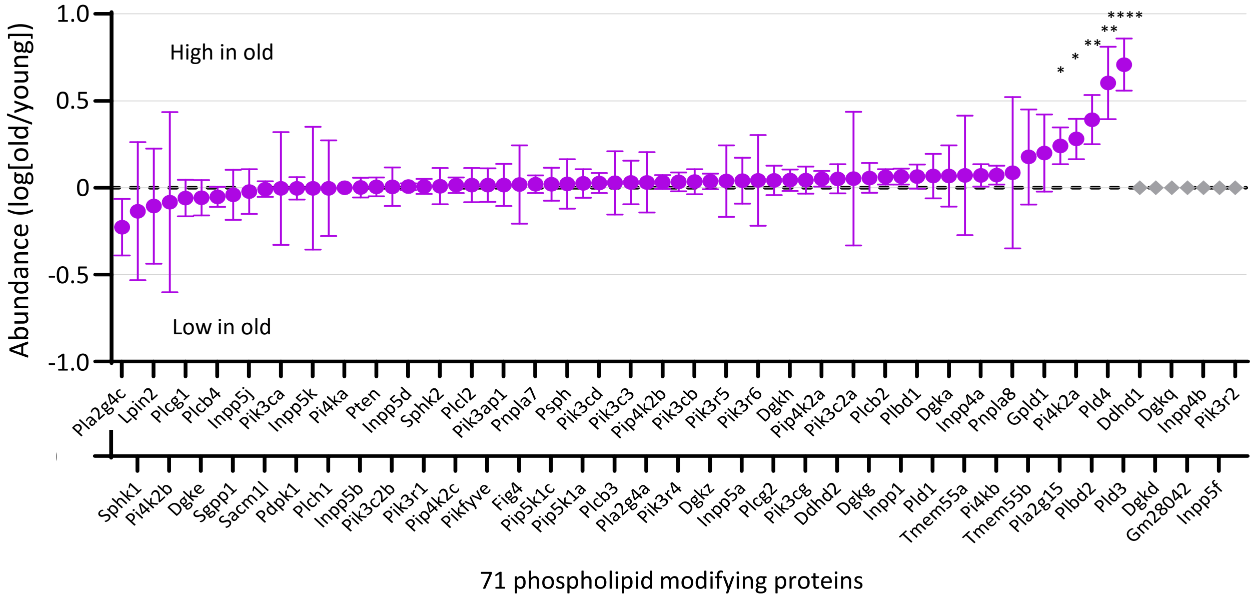

B

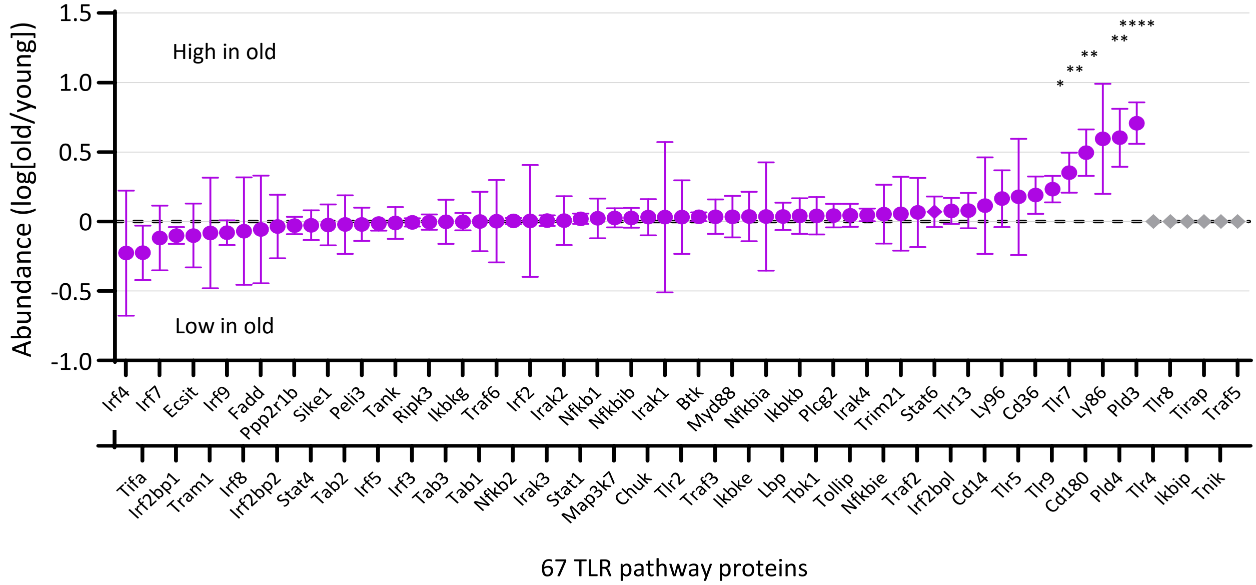

Supplemental Figure 7. Hornigold K *et al.* Altered phospholipid-modifying and TLR pathway protein expression with ageing

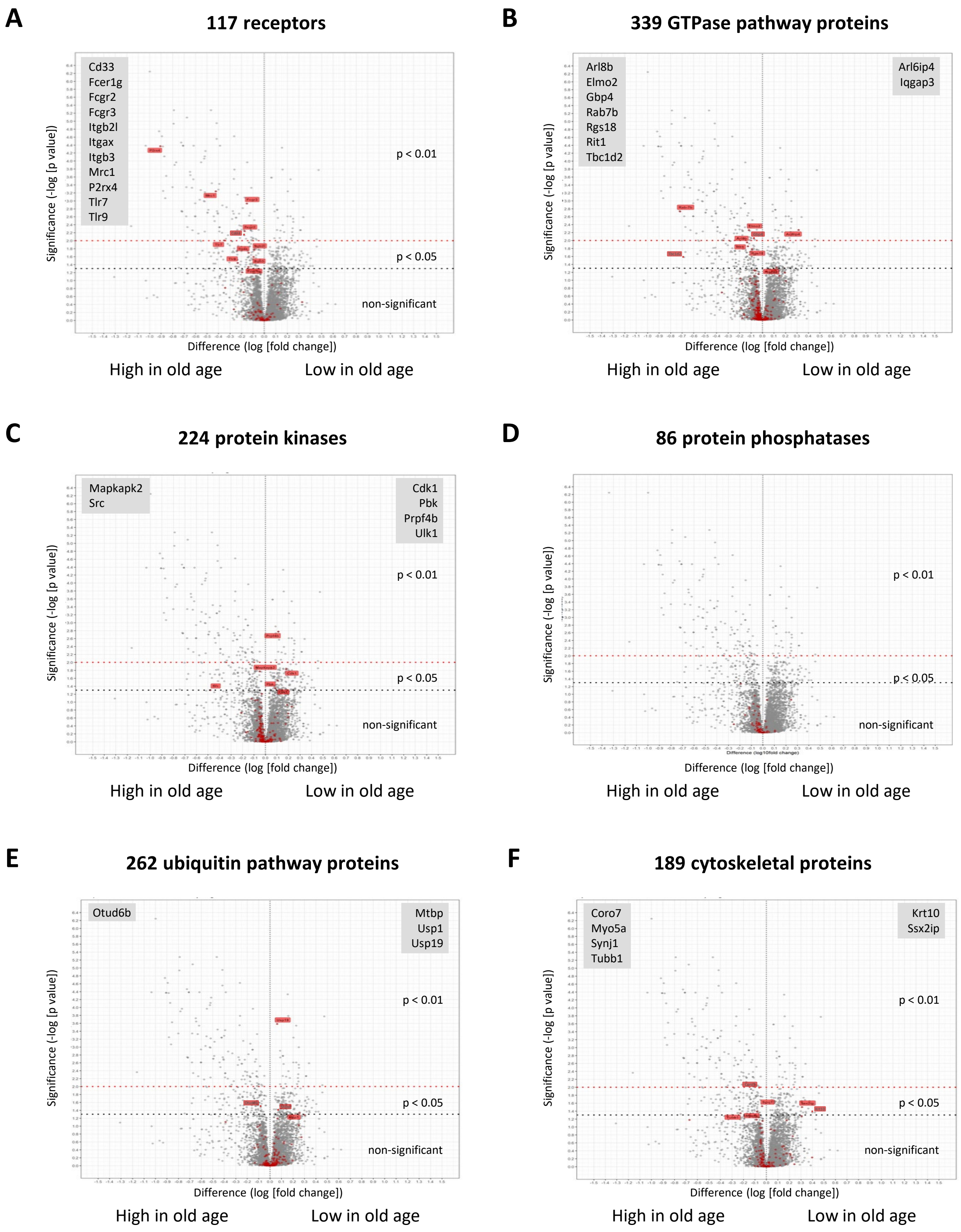

Supplemental Figure 8. Hornigold K *et al.* Altered expression of various neutrophil protein groups in old age
